# Potential Rad54 separation of function mutation highlights unique roles during homologous recombination

**DOI:** 10.1371/journal.pgen.1012136

**Published:** 2026-04-27

**Authors:** Jingyi Hu, David Moraga, Amanda Xu, Lauren Peysakhova, J. Brooks Crickard

**Affiliations:** Department of Molecular Biology and Genetics, Cornell University, Ithaca, New York, United States of America; SOM University of Pennsylvania: University of Pennsylvania Perelman School of Medicine, UNITED STATES OF AMERICA

## Abstract

Homologous recombination (HR) is a DNA repair pathway that utilizes a template-based approach to repair double-strand breaks within the genome. Template use requires the exchange of individual DNA strands, which members of the RecA family of recombinases facilitate. Rad51 is a primary strand exchange factor in eukaryotes. During regular mitotic DNA repair, Rad51 is aided by the DNA translocase Rad54, which acts as a motor to remodel the template DNA and stabilize primary-strand exchange intermediates. The regulation of this activity remains incompletely understood. Here, we have identified a conserved site within the C-terminal region of Rad54. The mutation of this site creates a separation of function at early strand-exchange intermediates *in vivo*. Using this mutant protein, we identify a novel intermediate essential for stabilizing displacement loop (D-loop) structures. This precedes the removal of Rad51 and DNA extension. Based on our experiments, we hypothesize that this Rad54 mutant cannot stabilize Rad51-mediated strand-exchange intermediates due to slippage during translocation, leading to failure in DNA remodeling. Identifying a mutant that disrupts this intermediate before Rad51 removal unifies existing models of Rad54-mediated D-loop formation and extension.

## Author summary

Homologous recombination (HR) is a DNA double-strand break repair pathway that functions at DNA breaks and stalled or collapsed replication forks. Rad51 is a critical enzyme that mediates replication fork protection and DNA strand exchange during HR. Rad51 is aided by the DNA motor protein Rad54, which can stabilize critical strand-exchange intermediates and remove Rad51 bound to dsDNA. Whether these Rad54 activities are the same or distinct has not been determined *in vivo*. Here, we have identified a Rad54 separation of function mutant that can remove Rad51 from dsDNA but fails to form primary-strand exchange intermediates *in vivo*. From this, we characterize the consequences of this mutation on HR outcomes. We also demonstrate, using single-molecule analysis, that this separation of function mutant has reduced affinity for dsDNA, thereby preventing stabilization of primary strand exchange intermediates.

## Introduction

Homologous recombination (HR) is a DNA double-strand break repair (DSBR) pathway that uses a homologous template to prime DNA synthesis to repair breaks [1–5]. In this pathway, ssDNA guides are formed by resection of the two ends of broken DNA [6–9]. This DNA is known as the recipient DNA because it will receive information during the recombination reaction. Filaments of the RecA family of recombinases form on the recipient DNA [10–13]. The filament guides a search of the genome for a matching DNA sequence [11,14–18]. Once a suitable donor homology is identified, the recombinase filament initiates a strand exchange reaction that generates a three-stranded DNA joint known as a displacement loop (D-loop) [12,19,20]. DNA polymerase can then initiate synthesis from the 3’ end of the recipient DNA and restore any lost information.

In the classic double-strand break repair pathway, resolution of DNA joints formed during HR can result in non-crossover outcomes (NCO) or crossover outcomes (CO). These outcomes are defined by the extent of genetic exchange between the donor and recipient, with CO outcomes resulting in greater exchange between the two DNA molecules [1,4]. Resolution can also occur through synthesis-dependent strand annealing (SDSA). This pathway results in apparent NCO outcomes when the second end of the break is located and used to finish the repair. When the second end of the break is not located, SDSA can transition to break-induced replication (BIR). This outcome can be extremely mutagenic, leading to complex genomic rearrangements and mosaic repair [21–23].

In eukaryotes, the mitotic RecA homolog is known as Rad51 [24,25], and during the homology search and subsequent transition to DNA synthesis, Rad51 is assisted by the DNA motor protein Rad54 [26–32]. The basic biochemical properties of Rad54 are that it hydrolyzes ATP to physically move along double-stranded DNA by tracking the minor groove in the 3’-5’ direction [33]. Multiple copies of Rad54 can function as single units, resulting in high processivity levels [27,29,32]. The primary role of Rad54 is to act as an accessory cofactor for Rad51, and interactions between these proteins increase the ATP hydrolysis activity of Rad54 by 3–5-fold [29,34,35]. The activities of Rad54 include the regulation of Rad51 at stalled or collapsed DNA replication forks [36], the removal of excess Rad51 that is pathogenically bound to dsDNA [37,38], the remodeling of nucleosomes [39–42], driving branch migration [35,43–46], collaboration with Rad51 to catalyze the formation of D-loops [19,29,32,47–49], and to grant accessibility of the 3’ end of the recipient DNA to DNA polymerase [48,50].

*In vivo*, Rad54 can promote Rad51 recombination activity through multiple mechanisms. One of these involves the removal of Rad51 from dsDNA and its stabilization on ssDNA [28,37,51–54], which aids in the maturation of recombinase filaments. Evidence for this stems from work involving the Rad51 Walker A mutant, Rad51K191R, which is deficient in Rad51 filament formation *in vivo* [55]. The overexpression of Rad54 can suppress the defect in this mutant, but only in the presence of Rad55/57 [56]. Rad55/57 are required for efficient Rad51 filament formation and counteracting the effects of the Srs2 helicase [57–61], whose primary function during HR is to remove Rad51 from ssDNA [62–67]. During Rad51 filament maturation, Rad54 can act by increasing the available pools of Rad51 and by physically protecting Rad51 filaments from Srs2 activity [54], regulating a dynamic equilibrium of Rad51 binding.

This model has been extended to support the hypothesis that the removal of Rad51 from newly paired dsDNA at strand-exchange intermediates is how Rad54 stabilizes the three-stranded D-loop structures [48]. This hypothesis explains the generation of an accessible 3’ end for further DNA extension, promoting repair and intermediate stability. A key feature of this model is that there is no functional separation of Rad54 activity, and control of Rad51 equilibrium is the only activity of Rad54. A contrasting model posits that Rad54’s activities are distinct functions, and that its role in forming D-loops does not require the removal of Rad51 from dsDNA [47]. The basis for this hypothesis is that Rad54 generates underwound DNA during translocation, and that underwound DNA is a more efficient substrate for recombination [29,47,68]. Whether D-loop stabilization and Rad51 removal from dsDNA are distinct or overlapping functions of Rad54 remains unclear *in vivo*.

Here, we investigated potential phosphorylation sites on the Rad54 protein and identified a specific residue in the C-terminal region that modulated Rad54 activity. Mutation of this residue yielded a separation of function mutant. We showed that the substitution of Aspartic acids for two Serine residues disrupted a bridging contact between the two RecA lobes of Rad54. Disrupting this contact caused severe defects in D-loop formation *in vivo.* This mutant was able to promote D-loop extension and the removal of Rad51 from dsDNA. Our data implies the identification of a novel physiological intermediate during D-loop formation and suggests that this step likely stabilizes the early D-loop. The stabilization of primary strand exchange intermediates potentially results in more stable late phase intermediates, impacting recombination outcomes. We hypothesize that stabilization is facilitated by Rad54 mediated loop extrusion.

## Results

**Fig 1:**
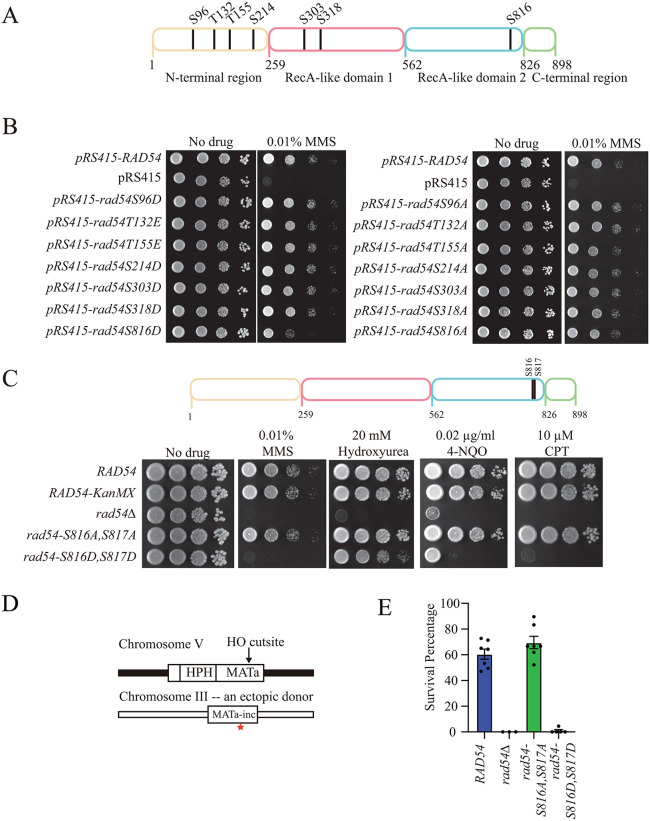
Screen of potentially phosphorylated residues in Rad54. (A). Schematic of residues in Rad54 that were identified as phosphorylated in the SuperPhos database. **(B).** Yeast complementation spot assay to monitor the effect of phosphomimic mutants (Left) and Alanine mutants (Right) of Rad54 at 0.01% MMS. **(C).** Serial dilution spot assay to monitor the impact of *rad54-S816A, S817A*, and *rad54-S816D, S817D* on sensitivity to 0.01% MMS, 20 mM Hydroxyurea (HU), 0.02 µg/ml 4-NQO, and 10 µM CPT. **(D).** Schematic diagram illustrating the substrate for an ectopic recombination assay. An HO endonuclease site is located on chromosome V, and the repair template is located on chromosome III. **(E).** Graph representing the survival percentage for *RAD54, rad54∆, rad54-S816A, S817A, and rad54-S816D, S817D*. The bars represent the mean, and the error bars represent the standard error measurements of at least seven independent experiments.

We identified potential phosphorylation sites in *Saccharomyces cerevisiae* Rad54 using the SuperPhos database [69]. Seven sites were selected, and these residues were mutated to Aspartic acid or Alanine for Serine, and to Glutamic acid or Alanine for Threonine. We performed complementation assays in *rad54∆* strains using methyl methanesulfonate (MMS) as a DNA-damaging agent (Fig 1A). Most of the mutated residues successfully complemented the MMS sensitivity phenotype. The exception was that the substitution of Aspartic acid for Serine at residue 816, which failed to fully complement the MMS sensitivity of the *rad54∆* strain (Fig 1B).

Interestingly, this site was identified as phosphorylated in two separate proteomic studies [69,70]. Both experiments were SILAC experiments that identified enrichment of Rad54 S816 phosphorylation. The first found enrichment in strains treated with 4-Nitroquinoline Oxide (4-NQO) after synchronization in the G1 phase of the cell cycle [69]. The second study was designed to determine which phosphorylation marks are affected by GCN2 deletion [70]. GCN2 is a kinase that globally regulates the G1/S checkpoint and the integrated stress response [71]. Both studies suggest that phosphorylation may occur during the G1 phase of the cell cycle when HR is typically repressed. This observation is consistent with our observation that the *rad54-S816D* allele shows increased MMS sensitivity and suggests that phosphorylation may be a repressive mark.

After identifying this repressive amino acid substitution, we chose to characterize how this mutation negatively impacted Rad54’s function during the HR reaction and sought to use it to further our understanding of Rad54 mediated D-loop formation *in vivo*. We inspected the S816 site and found that the adjacent residue was also a Serine (Fig 1C). We speculated that mutating both residues may result in a more severe phenotype. We generated *rad54-S816A, S817A,* and *rad54-S816D, S817D* substitutions and tested them for complementation. We observed an enhanced phenotype in the *rad54-S816D, S817D* strain (S1A Fig). There was no observable phenotype when only the S817 was changed to an Aspartic acid residue (S1A Fig).

Under the MMS conditions used for the initial screen, *rad54-S816D, S817D* was as severe as the *rad54∆* strain. Therefore, we tested whether this mutation could be expressed at similar levels to RAD54 and whether Rad54 foci formed in response to MMS treatment. We monitored *RAD54-GFP*, *rad54-S816A, S817A-GFP,* and *rad54-S816D, S817D-GFP* for expression with and without MMS (S2A Fig). All three of these proteins were expressed at similar levels with and without MMS and formed Rad54 foci in response to MMS treatment (S2ABC Fig). From this, we concluded that there was no defect in the expression or stability of these Rad54 mutants.

Different DNA-damaging agents utilize distinct repair pathways. We evaluated whether *rad54-S816D, S817D* was sensitive to other types of damaging reagents. Hydroxyurea (HU) is a drug that depletes nucleotide pools and acts as a damaging agent by inducing replication stress [72]. We observed that *rad54∆* strains are unable to grow in the presence of 20 mM HU (Fig 1C). In contrast, we observed only a minor growth defect in *rad54-S816D, S817D,* suggesting that this protein retains partial function and is relatively unaffected at lower HU concentrations.

Camptothecin (CPT) is a topoisomerase inhibitor that creates protein-DNA adducts [73]. These adducts are primarily repaired via cross-link repair pathways. However, at sufficiently high concentration, these adducts cause transcription and replication stress that requires HR for repair. We found that *rad54-S816D, S817D* could not rescue the CPT phenotype (Fig 1C). Finally, we tested sensitivity to 4-Nitroquinoline Oxide (4-NQO). This drug is a UV-mimetic chemical that forms base adducts [74]. At sufficiently high concentrations, it can lead to transcription- or replication-induced breaks. Treatment with 4-NQO resulted in sensitivity in the *rad54-S816D, S817D* strains (Fig 1C). From this, we conclude that *rad54-S816D, S817D* retain partial function under some DNA-damaging conditions, depending on the type of damage.

After testing sensitivity to DNA damaging agents, we evaluated the competency of *rad54-S816D, S817D* in repairing a single double-strand DNA break at an ectopic donor site. We used a reporter assay with a *MATa* locus containing an HO endonuclease cleavage site on chromosome V (Fig 1D) [75]. A homologous non-cleavable *MATa-inc* is located on chromosome III (Fig 1D). Upon induction with galactose, the DNA is cleaved, and repair occurs using the ectopic allele. Cells will only survive if they can repair the break. We measured the survival percentage after induction of a break. In wildtype (WT) strains, the survival frequency was 60–65% (Fig 1E). This outcome was also the case with the *rad54-S816A, S817A* strains (Fig 1E). In the *rad54∆* strain, there is no survival. The *rad54-S816D, S817D* strains exhibited only ~1% survival (Fig 1E). From this, we conclude that the *rad54-S816D, S817D* is deficient in repairing a single double strand break from an ectopic site.

**Fig 2:**
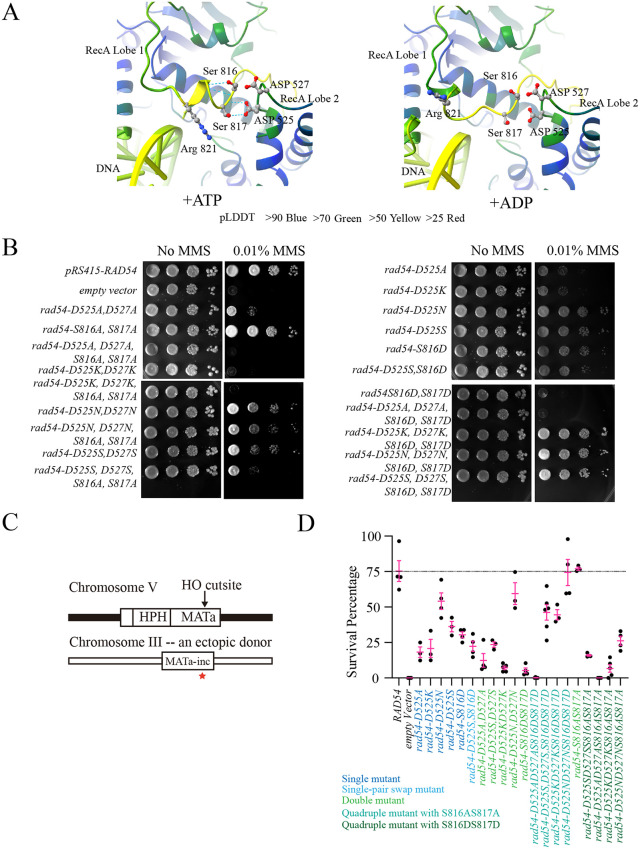
Mutations in Rad54 are separation-of-function mutants. **(A).** Alphafold3 structural predictions for Rad54 + dsDNA + ATP (Left) and Rad54 + dsDNA + ADP (Right). Colors of the structure reflect the confidence in the prediction. The pLDDT values are listed below the structures. **(B).** Serial dilution spot assays to test complementation of *rad54∆* with *pRS415-RAD54, Empty vector, rad54-D525A, D527A, rad54-D525K, D527K, rad54-D525N, D527N, rad54-D525S, D527S, rad54-S816A, S817A, rad54-D525A, D527A, S816A, S817A, rad54-D525K, D527K, S816A, S817A, rad54-D525N, D527N, S816A, S817A, rad54-D525S, D527S, S816A, S817A, rad54-D525A, rad54-D525K, rad54-D525N, rad54-D525S, rad54-S816D, rad54-D525S, S816D, rad54-S816D, S817D, rad54-D525A, D527A, S816D, S817D, rad54-D525K, D527K, S816D, S817D, rad54-D525N, D527N, S816D, S817D, and rad54-D525S, D527S, S816D, S817D* for MMS sensitivity. **(C).** Schematic diagram illustrating the substrate for an ectopic recombination assay. An HO endonuclease site is located on chromosome V, and the repair template is located on chromosome III. **(D).** Graph illustrating percentage survival for complementation of *rad54∆* with *pRS415-RAD54, Empty vector, rad54-D525A, D527A, rad54-D525K, D527K, rad54-D525N, D527N, rad54-D525S, D527S, rad54-S816A, S817A, rad54-D525A, D527A, S816A, S817A, rad54-D525K, D527K, S816A, S817A, rad54-D525N, D527N, S816A, S817A, rad54-D525S, D527S, S816A, S817A, rad54-D525A, rad54-D525K, rad54-D525N, rad54-D525S, rad54-S816D, rad54-D525S, S816D, rad54-S816D, S817D, rad54-D525A, D527A, S816D, S817D, rad54-D525K, D527K, S816D, S817D, rad54-D525N, D527N, S816D, S817D, and rad54-D525S, D527S, S816D, S817D* in an ectopic recombination assay. The bars represent the mean, and the error bars represent standard error measurements of at least three independent experiments.

We next evaluated whether there was a structural basis for the defect in Rad54 activity caused by substituting Aspartic acid for Serine at positions 816 and 817. We initially used the existing *Danio rerio* Rad54 crystal structure [76] to evaluate the position of these residues. However, we could not find an interaction that may be significantly disrupted, leading to enzyme inactivation. We then used AlphaFold3 [77] to predict the structure of yeast Rad54 in the presence and absence of dsDNA (S3AB Fig). The inclusion of dsDNA in the prediction led to a conformational change in the Rad54 molecule that repositioned the S816 and S817 residues to potentially form an interaction with residues D525 and D527 on the adjacent RecA lobe of Rad54 (Figs 2A and S3AB). It should be noted that S816 and S817 are in an unstructured region of the protein, and AlphaFold has limited confidence in their positioning. However, the movement in the prediction suggested a mobile region of the protein and generated a testable hypothesis.

We further predicted the same Rad54 structures with ADP instead of ATP. The unstructured region containing Rad54 S816 and S817 underwent a significant conformational change in the presence of ADP (Figs 2A and S3AB). This led to the repositioning of a third residue, Arginine 821, which interacts with dsDNA in the ATP-bound form, away from the DNA (Fig 2A), suggesting that S816 and S817 are important during DNA translocation. By using ChimeraX [78] we predicted hydrogen-bonding patterns for S816, S817, D525, and D527. Predictions in ChimeraX are based on the distance between the hydrogen-bond donor and acceptor. The S816 residue formed a hydrogen bond with a residue C-terminal to itself within the same RecA lobe. However, D525 formed an interaction that extended across the gap between the RecA lobes, involving S817 and the peptide backbone (Fig 2A). Importantly, these interactions were lost in the ADP bound form of the enzyme (Fig 2A). While these models are only predictive, they allowed us to develop a hypothesis that the S816 residue may participate in a critical interaction during the conformational changes associated with ATP hydrolysis.

To systematically test this model, we generated four mutant groups. The first group was single-site substitutions at D525. Substitutions included a D525A, a charge swap D525K, and two conservative mutations, D525N and D525S. These amino acid substitutions were maintained throughout the structure function analysis. The mutants were tested using both complementation of MMS sensitivity (Fig 2B) and survival in an ectopic repair assay (Fig 2C and 2D). As predicted, the D525A and D525K mutants failed to complement the MMS phenotype and resulted in poor survival 75% (WT) versus 15–20% (D525A, D525K p = 0.0016 and p = 0.003, respectively) in the ectopic recombination assay. The conservative mutations D525N and D525S were more efficient at complementation in the MMS experiment and showed higher survival rates in the ectopic repair assay. However, they remained defective for full complementation (Fig 2D). We further tested the ability of the D525A and D525K substitutions to complement HU, 4-NQO, and CPT (S4A Fig). Like the *rad54-S816D, S817D* allele, the *rad54-D525A* and *rad54-D525K* alleles were able to complement sensitivity to HU and showed sensitivity to CPT and 4-NQO (S4A Fig).

The second group of mutants we tested was double mutants with the D525 position. We made a site-swap mutant, converting the Aspartic acid at 525 to Serine and the Serine at 816 to Aspartic acid. This substitution did not suppress the MMS phenotype and showed poor survival in the ectopic repair assay (Fig 2B and 2D). The double mutant was not significantly different from the *rad54-S816D* single mutant (p = 0.10). The failure of this swap to suppress the S816D phenotype suggested that the stabilizing interaction is more complex than a single amino acid interaction. Our model predicted potential contributions from S817 and D527. These residues are less conserved in higher eukaryotes (S5A Fig), but they may still participate in the interaction according to the AlphaFold model. Therefore, we made double mutants at the 525 and 527 positions. We tested D525A,D527A, D525S,D527S, D525K,D525K, and D525N,D525N (Fig 2B and 2D). Except for the D525K,D527K substitution, the double mutants were not significantly different from the single substitutions at 525. These data suggest that these two residues likely participate in the same interaction, but there is no additional defect in mutating the D527 position.

The *rad54-S816A, S817A* substitution fully complemented MMS sensitivity, and we next made D525, D527 mutations in the *rad54-S816A, S817A* allele. The *rad54-D525A, D527A, S816A, S817A* allele completely failed to complement the MMS phenotype and completely failed to repair any breaks in the ectopic recombination assay (Fig 2B and 2D), representing a completely dead mutant. The substitution of Lysine, Serine, or Asparagine at D525 and D527 in the *rad54-S816A, S817A* mutants was all significantly worse than the *rad54-S816A, S817A* mutant itself in both the MMS complementation and the ectopic recombination assay (Fig 2B and 2D). Interestingly, the conservative mutations of Serine and Asparagine performed better than Lysine, matching the trend observed with other mutant groups, indicating the importance of potential for hydrogen bond formation between D525, D527, and S816, S817.

Finally, we tested this series of mutants to determine whether we could suppress the phenotypes associated with the *rad54-S816D, S817D* mutant. We found that the *rad54-D525A, D525A, S816D, S817D* mutants failed to complement MMS sensitivity or ectopic repair survival (Fig 2B and 2D). However, Lysine, Serine, or Asparagine at positions 525 and 527 suppressed the *rad54-S816D, S817D* phenotypes (Fig 2B and 2D). In the ectopic repair assay, Lysine and Serine substitutions resulted in partial complementation (>10-fold recovery), and Asparagine resulted in full complementation. Interestingly, this is the only group in which the Lysine substitutions performed equivalently to the Serine substitutions. This is likely due to the charge on the S816D, S817D substitutions and the restoration of a strong interaction between the RecA lobes. From this, we conclude that stabilization of the interaction between the two RecA lobes is essential for Rad54 function. The interaction likely occurs through a hydrogen-bonding network between the amino acid side chains and the peptide backbone, which may stabilize the ATP-bound form of the enzyme. While we did not physically show linkage between these sites, our data suggests a functional interaction that is important double strand break repair.

### *rad54-S816D, S817D* is defective in stable D-loop formation

**Fig 3:**
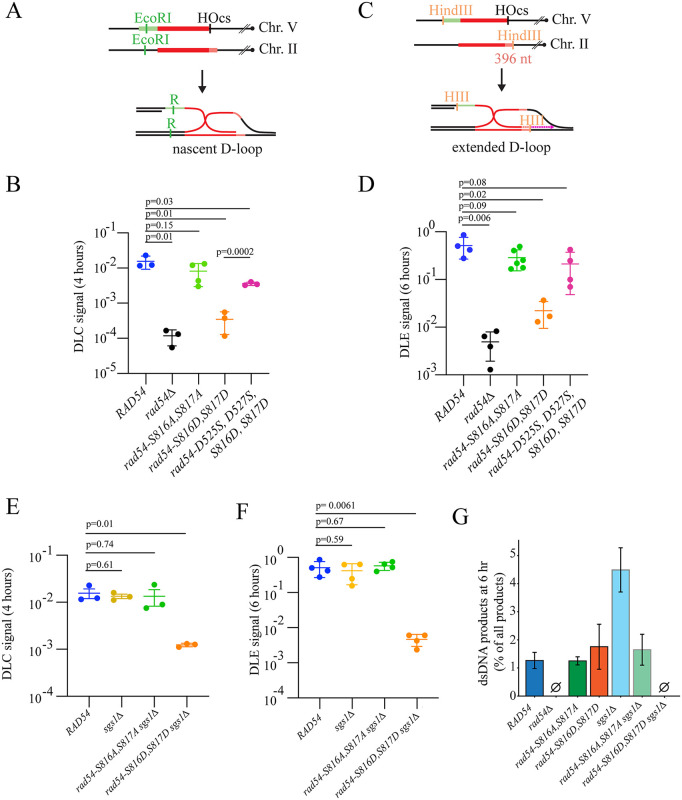
Mutations in *RAD54* impact recombination intermediates. **(A)**. Schematic diagram illustrating the D-loop capture assay to trap the formation of nascent D-loops during recombination. **(B).** Graph representing D-loop capture efficiency at 4 hours post-break induction for *RAD54*, *rad54∆*, *rad54-S816A, S817A*, *rad54-S816D, S817D*, and *rad54-D525S, D527S, S816D, S817D.* The bar represents the mean, and the error bars represent the standard deviation for at least three independent experiments. **(C).** Schematic diagram illustrating the assay to monitor D-loop extension. **(D).** Graph representing D-loop extension at 6 hours for *RAD54*, *rad54∆*, *rad54-S816A, S817A*, *rad54-S816D, S817D*, and *rad54-D525S, D527S, S816D, S817D.* The bar represents the mean, and the error bars represent the standard deviation for at least three independent experiments. **(E).** Graph representing D-loop capture efficiency at 4 hours post-break induction for *sgs1*∆, *rad54-S816A, S817A sgs1∆*, *rad54-S816D, S817D sgs1∆*. The bar represents the mean, and the error bars represent the standard deviation for at least three independent experiments. **(F).** Graph representing D-loop extension at 6 hours for hours post-break induction for *sgs1*∆, *rad54-S816A, S817A sgs1∆*, *rad54-S816D, S817D sgs1*∆. The bar represents the mean, and the error bars represent the standard deviation for at least three independent experiments. **(G).** The percentage of dsDNA among total extension products for *RAD54*, *rad54∆*, *rad54-S816A, S817A*, *rad54-S816D, S817D*, *sgs1∆*, *rad54-S816A, S817A sgs1∆*, *rad54-S816D, S817D sgs1∆*. The error bars represent the standard error measurement for at least three independent experiments.

To identify the repair intermediate at which the *rad54-S816D, S817D* strain is defective, we used a previously established D-loop capture assay [14,19,79,80]. This assay also employs an ectopic repair site within the genome and can be used to isolate nascent and extended D-loops. These DNA structures represent the initial strand exchange intermediates. For nascent D-loop capture, after induction of a break using the HO endonuclease, the cells are cross-linked with psoralen. Cross-linking traps the nascent D-loops and prevents dissociation during purification. The efficiency of D-loop capture is then quantified by isolating genomic DNA, oligo hybridization, proximity ligation, and quantifying the amount of isolated DNA by qPCR (Figs 3A and S6AB). We monitored D-loop capture at 4 hours, and as expected, the *rad54∆* strains had a signal that was 100-fold lower than the WT. This value was also the same as that obtained from qPCR performed without the oligo to restore restriction enzyme sites, indicating this is the background (S6C Fig). The *rad54-S816A, S817A* allele was slightly lower than WT. However, this difference was not significant (Fig 3B). The *rad54-S816D, S817D* strain was 47-fold lower than WT (Figs 3B and S6CDEFG), indicating a severe defect in D-loop capture. To further validate this phenotype, we performed additional D-loop capture experiments using the *rad54-D525S, D527S, S816D, S817D* allele, which represents one of the intragenic suppressors identified in the complementation analysis. This mutant suppressed the D-loop capture phenotype, increasing capture efficiency approximately 10-fold and making it only 5-fold less efficient than WT (Fig 3B). The observation is consistent with the ectopic repair survival experiments.

The extension of D-loops stabilizes the nascent three-strand intermediates. D-loop extension from the newly paired 3’ end can be measured using the same strains, but without psoralen cross-linking (Figs 3C and S7AB). A caveat to this analysis is that the D-loop must be extended by at least 400 nt to receive the second restriction site and be detected in the assay. Therefore, defects in extension could reflect low D-loop capture efficiency or premature disruption of DNA synthesis, resulting in shorter extension products. As with the D-loop capture assay, there was only a modest difference from WT in the ability of *rad54-S816A, S817A* to extend D-loops, and this was not significant. However, there was a significant loss (20-fold, p = 0.02) of D-loop extension in the *rad54-S816D, S817D* strain at 6 hours (Figs 3D and S7CDEF). This change is still better than the 100-fold loss in the *rad54∆* strain. The *rad54-S816D, S817D* phenotype was again suppressed in the *rad54-D525S, D527S, S816D, S817D* strain (Figs 3D and S7CDEF). From this, we conclude that the interaction between S816 and D525 is required for efficient D-loop capture and stabilization.

Sgs1 is a DNA helicase that participates in DNA end-resection, reversal of toxic recombination intermediates, and dissolution of Holliday junctions [81]. Sgs1 is also implicated in the reversal of D-loops [19,79,82]. We reasoned that if *rad54-S816D, S817D* were subjected to a high level of D-loop reversal, then deletion of *SGS1* might suppress most of the observed defects in D-loop capture. However, this was not the case, and only a small increase in D-loop capture was observed in the *rad54-S816D, S817D sgs1∆* double mutant strain. Capture efficiency remained 20-fold lower than WT (Figs 3E and S6CDEFG). The increase observed in the *rad54-S816D, S817D sgs1∆* double mutants translate into a 3-fold increase in capture efficiency compared with the *rad54-S816D, S817D* single mutant. Previously, it has been shown that *SGS1* has a stronger impact on D-loop capture at earlier time points, and analysis at 4 hours may contribute to the lack of observable phenotype.

There was no observable defect in the amount of D-loop extension for the *sgs1∆* or the *rad54-S816A, S817A sgs1*∆ strains (Fig 3F). Surprisingly, in the *rad54-S816D, S817D sgs1∆* strain, there was now a 100-fold loss in D-loop extension. This observation suggests that while D-loop capture was slightly improved, there was insufficient extension to reach the second restriction site (Fig 3F). This observation means that the *rad54-S816D, S817D* strains are competent for D-loop extension if the primary D-loop is stabilized, because there is a significant (5-fold) reduction in extension between the *rad54-S817D, S817D* and the *rad54-S817D, S817D sgs1∆* strains (Fig 3F) This extension is lost in the *rad54-S816D, S817D sgs1∆* mutant, indicating a synthetic genetic interaction between *RAD54* and *SGS1*.

In this assay, one side of the double-strand break lacks homology to the target. The lack of homology prevents repair through SDSA or classical DSBR, making this system a break-induced replication (BIR) system. In BIR based repair, DNA synthesis proceeds through a migrating bubble, using conservative replication [23,83]. The newly extended ssDNA is used as a template for second strand synthesis [83,84]. Therefore, the extended D-loops can also be characterized by their ssDNA-to-dsDNA content (Figs 3G and S7F). The amount of dsDNA is measured by not adding a hybridizing oligo during DNA extraction, and comparing this to the same conditions with an oligo added. The addition of an oligo restores a restriction site that is used to cut the DNA. If DNA is cut in the absence of the oligo, it means that the restriction site has been restored by natural DNA synthesis. The amount of restriction site cutting and re-ligation with and without oligo can then be used to compare the ssDNA to dsDNA content, reflecting the amount of second strand synthesis [19,80]. This value is low in WT but is elevated 3–4-fold in *sgs1∆* strains [19]. We observed this same increase in dsDNA in a *sgs1∆* strain (Figs 3G and S7F). Surprisingly, this increase was lost in the *rad54-S816A, S817A sgs1∆* strain (Fig 3G). This result suggests a genetic interaction between *RAD54* and *SGS1* during the repair synthesis phase, and a slight defect in the *rad54-S816A, S817A* mutant. It should be noted that the extension values for *rad54∆* and *rad54-S816D, S817D sgs1∆* were so low that this type of analysis could not yield reliable results.

Our data suggested that D-loop extension was less effected than D-loop capture in the *rad54-S816D, S817D* strain. This would imply that *rad54-S816D, S817D* may be competent to remove Rad51 from dsDNA. To test this genetically, we overexpressed *RAD51* under a galactose-inducible promoter. This assay has been used to show that both *RAD54* and its paralog, *RDH54*, can remove Rad51 from dsDNA [37,85]. The phenotype is weak in *rad54∆* strains and is significantly stronger in *rdh54∆* strains. However, the effects of *RAD54* in this assay are pleiotropic with *rdh54∆*. Therefore, the weak phenotype associated with *rad54∆* is the best metric to determine if our *rad54* alleles could rescue this phenotype. In these assays, overexpression of Rad51 does not result in cell death and is reversible if the plasmid overexpressing Rad51 is lost. However, persistent overexpression of can lead to chromosome loss [37]. These phenotypes are caused by extensive binding of Rad51 to chromosomes, even in the absence of DNA repair.

We found that both *rad54-S816A, S817A* and *rad54-S816D, S817D* behaved like WT in the RAD51 overexpression experiments (S8A Fig). To enhance the phenotype, we also overexpressed a mutant *RAD51* allele, *rad51-I345T*, which binds ds- and ssDNA more tightly [58]. We expected this would be a greater challenge to remove from the dsDNA and may expose any weak phenotypes associated with the *rad54* mutants. Overexpression of *rad51-I345T* did slightly strengthen the overexpression phenotype in the *rad54∆*. However, both the *rad54-S816A, S817A* and *rad54- S816D, S817D* alleles were able to remove Rad51 from dsDNA (S8A Fig). Despite the weak phenotype in this experiment, our data are consistent with an enzyme that can remove Rad51 from dsDNA but is deficient in D-loop formation.

### Interhomolog allelic recombination

**Fig 4:**
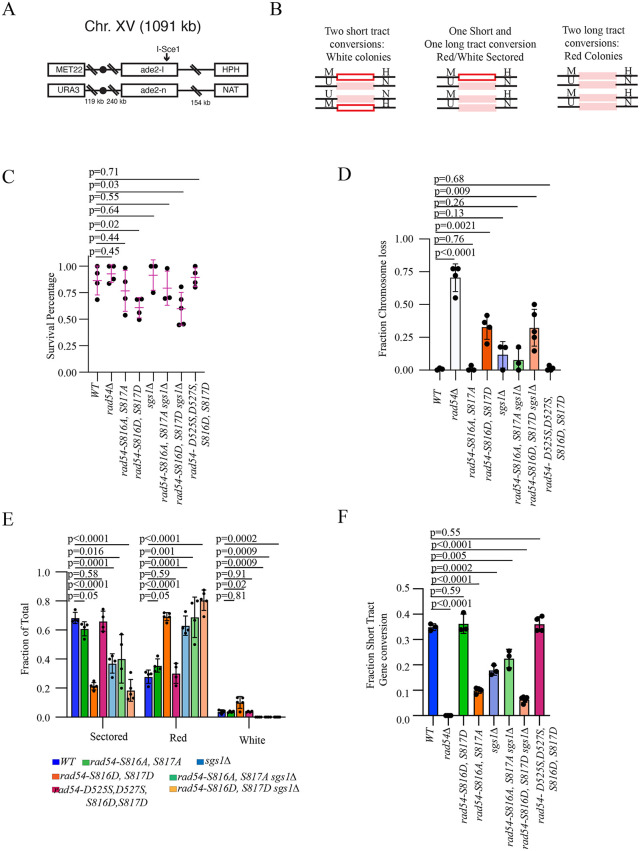
Impact of *RAD54* mutations on allelic recombination between homologous chromosomes. **(A).** Schematic diagram illustrating the DNA reporter used to analyze the effect of Rad54 mutation on allelic recombination. **(B).** Schematic diagram illustrating the potential gene conversion outcomes and HR based repair pathways during allelic recombination. **(C).** Graph representing the plating efficiency of strains treated with galactose. These were measured by dividing the number of colonies formed after galactose treatment to a no-galactose control. The bar represents the mean of the data, and the error bars the standard deviation of at least 3 independent experiments. **(D).** Graph representing the fraction of colonies that undergo chromosome loss for All strains tested. The bar represents the mean and the error bars the standard deviation of at least four independent experiments. **(E).** Graph representing the percentage of solid red, solid white, and sectored colonies for all strains tested. The bar represents the mean of the data, and the error bars represent the standard deviation of at least three independent experiments. **(F).** Graph representing the fraction colonies that undergo short tract gene conversion for all strains tested. This data was generated by measuring the colonies that grew on YNB (–Ade) + dextrose. The bar represents the mean, and the error bars the standard deviation of at least four independent experiments.

The *rad54-S816D, S817D* mutant had diminished D-loop capture and failed to promote survival during ectopic recombination. Ectopic recombination is generally less efficient than recombination between sister chromatids or homologous chromosomes and may be more sensitive to subtle changes in recombination efficiency [75]. Consequently, we investigated the impact of the *rad54-S816A, S817A,* and *rad54-S816D, S817D* alleles on recombination between homologous chromosomes. We used a reporter assay based on the *ADE2* gene in diploid *S. cerevisiae* [86–89]. Each copy of chromosome XV has a different inactive allele of *ade2*. One copy contains an *I-Sce1* nuclease site (*ade2-I*), and the other has an inactivating mutation located within the gene (*ade2-n*) (Fig 4A). Upon induction of the nuclease, a double strand break forms when one of the sister chromatids is cut. If this is repaired via the uncut sister, then the restriction site will be restored, and re-cutting can occur. Gene conversion and elimination of the restriction site occur when repair uses the homologous chromosome. Types of gene conversion events can be determined by reconstitution of the *ADE2* genes. If long tract repair occurs, then the gene conversion event will acquire the deleterious mutation from the homolog, and the yeast colony will appear red. If short tract repair occurs, the mutation will not be acquired, and a functional *ADE2* gene will be reconstituted, resulting in a white colony (Fig 4B). Each sister can be repaired via a long tract or short tract gene conversion event, and due to independent assortment, a colony can be red (two long tracts), white (two short tracts), or sectored (one long tract and one short tract).

We initially evaluated strains for survival after galactose treatment by comparing the plus and minus galactose conditions. We found that the WT, *rad54∆/rad54∆,* and *rad54-S816A, S817A* survived with 80–100% efficiency (Fig 4C). In contrast, the *rad54-S816D, S817D* mutant survived ~50–70% of the time (Fig 4C). However, this analysis was complicated in the *rad54∆/rad54∆ and rad54-S816D, S817D* strains because, after galactose treatment, the colonies formed a mixture of normal-sized and microcolonies. We hypothesized that the micro-colonies may represent colonies that have undergone chromosome loss. We tested this by replica-plating the colonies onto YNB (-Met) + dextrose and YP + Hygromycin + dextrose. The *MET22* and *HPHMX* markers are located on opposite sides of the centromere on chromosome XV (Fig 4A), and loss of both markers indicates a chromosome loss event. As expected, the microcolonies exhibited loss of both markers. In the *rad54∆/rad54∆* strains, ~ 70% of the surviving colonies exhibited loss of the cut chromosome. In WT and the *rad54-S816A, S817A* mutant <1% of colonies scored for chromosome loss (Fig 4D). In the *rad54-S816D, S817D* mutant ~30% of the population exhibited loss of the cut chromosomes (Fig 4D). Importantly, this value was reduced to <1% in the *rad54-D525S, D527S, S816D, S817D* intragenic suppressor mutant. From this, we conclude that the *rad54-S816D, S817D* results in a significant loss of the cut chromosome. In subsequent analyses of recombination outcomes, the microcolonies and chromosome loss events were excluded.

The normal size colonies observed in the *rad54∆/rad54∆* were red, and there were no sectored or white colonies. To determine if the red colonies were recombinants or uncut colonies, we replica plated cells on YNB (–Ade) + raffinose + galactose plates. If colonies are uncut, they will be forced to convert to *ADE2* to survive, resulting in white papillae in the red transferred colonies. If cells don’t survive, they have repaired *ade2* by long tract gene conversion or non-homologous end joining (NHEJ). In the *rad54∆/rad54∆* strains, this represented most of the large colonies. A limitation of this assay is the inability to distinguish between DSBs that may be uncut in the reinduction assay due to low-fidelity repair via non-homologous end joining (NHEJ). In fact, similar assays have shown that ~35% of repair events occur through Rad51-independent pathways in the absence of Rad51 [90]. Given the uncertainty of competition between Rad51-dependent and independent pathways, we have limited our interpretation to reflect that our observed changes only reflect a loss in gene conversion outcomes. Whether this occurs because of an increase in gene conversion tract length or an increase in competing repair pathways is unclear.

In our hands, sectored colonies are the most common outcome in WT, occurring approximately 65–70% of the time. This was also observed in the *rad54-S816A, S817A* mutant (Fig 4E). However, with the *rad54-S816D, S817D* allele, sectored colonies accounted for only about 20–25% of the population, with most colonies appearing solid red. The fraction of short tract gene conversion events can be measured by replica plating colonies on YNB (–Ade) + dextrose. Under these conditions, only colonies/sectors that had undergone short tract gene conversion could grow. In WT and *rad54-S816A, S817A,* this was ~ 35% of the population (Fig 4F). By contrast, this group was reduced to ~10% in *rad54-S816D, S817D* strains (Fig 4F**, p < 0.0001**). Importantly, this phenotype was fully suppressed by the *rad54-D525S, D527S, S816D, S817D* mutant (Fig 4F).

The helicase Sgs1 is known to shorten gene conversion tracts, and *sgs1∆* strains result in higher levels of long tract gene conversion [91]. From our previous experiments, we identified a novel genetic interaction between Rad54 and Sgs1. Therefore, we generated *SGS1* deletion strains with *RAD54*, *rad-54S816A, S817A*, and *rad54-S816D, S817D,* to further explore this interaction. Deletion of *SGS1* resulted in a moderate increase in chromosome loss (Fig 4D). However, these strains retained a significant amount of recombination (Fig 4F). There was no observable increase in chromosome loss or change in short tract gene conversion in the *rad54-S816A, S817A sgs1∆* double mutant. In the *rad54-S816D, S817D sgs1∆* double mutant, there were comparable levels of chromosome loss to the *rad54-S816D, S817D* strains (Fig 4D). However, there was a further decrease in short tract gene conversion (Fig 4F). This is further evidence of a genetic interaction between *RAD54* and *SGS1*.

The reporter strains used in these experiments also carry antibiotic markers downstream of the *ade2* genes. The segregation of these markers can be used to determine whether the recombination results in a Crossover (CO), Non-Crossover (NCO), or Break-induced Replication (BIR) outcome (Fig 4A and 4B). Outcomes can be scored by replica plating colonies onto YPD + Nourseothricin (clonNat) or YPD + Hygromycin (Hph). Colonies in which a sector grows on one selection, and the other sector grows on the other selection, can be designated as crossovers. This number is multiplied by two because for every observable crossover outcome, there is the potential for an unobservable crossover. BIR is scored for sectored colonies in which one sector grows under both selections, and the other sector only grows under Nat selection. For red colonies, a similar method can be used for scoring, but these can only be termed BIR-like and could result from other types of repair, as described above. Therefore, these outcomes should be interpreted cautiously.

We analyzed the *rad54-S816A, S817A*, and *rad54-S816D, S817D* strains for recombination outcomes. There was a small but significant increase in NCO between the WT and *rad54-S816A, S817A*, and there was no change in BIR outcomes (S9A Fig). However, there was a more significant drop in CO outcomes and an increase in NCO outcomes associated with the *rad54-S816D, S817D* (S9A Fig**, p < 0.0001**). We observed a small increase in BIR associated with this mutant (S9A Fig**, p = 0.04**). We evaluated the *rad54-D525S, D527S, S816D, S817D* rescue mutant, and this restored CO and NCO to WT levels (S9A Fig). There was no significant difference in outcomes between the WT and *sgs1∆ or rad54-S816A, S817A sgs1∆* strains (S9B Fig). However, in the *rad54-S816D, S817D sgs1∆* strain, there was a complete loss of CO (S9B Fig), and this was matched by an increase in NCO and a small increase in BIR events (S9B Fig). The complete loss of CO outcomes was the only significant change from the *rad54-S816D, S817D* strain. We have included this data in the manuscript, but due to our limited ability to differentiate the competition between Rad51-dependent and independent repair pathways, we have not offered an interpretation.

### Rad54 S816D/S817D is defective in binding dsDNA

**Fig 5:**
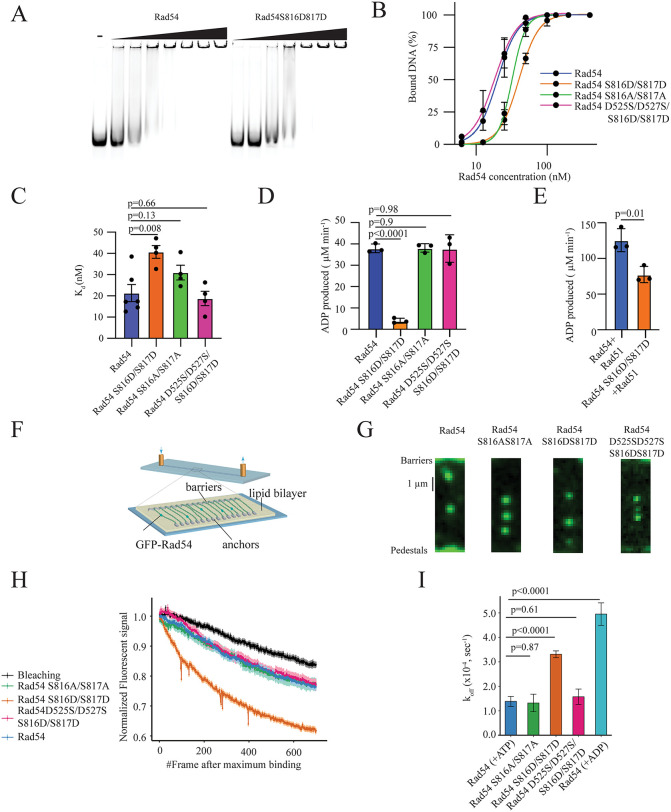
Rad54 S816D/S816D has reduced affinity for dsDNA. **(A).** Representative 0.5% TBE gel for EMSA with Rad54 and Rad54 S816D/S817D with a 90 bp Atto647N labelled dsDNA. **(B).** Graph representing the quantification of the EMSE data for Rad54, Rad54 S816A/S817A, Rad54 S816D/S817D, and Rad54 D525S/D527S/S816D/S817D. The data are fit by a hill equation. The error bars represent the standard error measurement of at least four independent experiments. **(C).** Graph representing the *K*_*d*_ measurements for individual EMSA experiments for Rad54, Rad54 S816A/S817A, Rad54 S816D/S817D, and Rad54 D525S/D527S/S816D/S817D. The bar represents the mean, and the error bars represent the standard error measurement of the experiment. **(D).** Graph representing the amount of ADP produced per minute in an ATP hydrolysis assay for Rad54, Rad54 S816A/S817A, Rad54 S816D/S817D, and Rad54 D525S/D527S/S816D/S817D. The bar represents the mean, and the error bars represent the standard deviation of four independent experiments. **(E).** Graph representing the amount of ADP produced per min for Rad54 and Rad54 S816D/S817D. The bar represents the mean, and the error bar represents the standard deviation of 3 independent experiments. **(F).** Schematic diagram illustrating DNA curtains experiments to test the activity of Rad54 on dsDNA. This image is reproduced through a CC-BY 4.0 license from [95]. **(G).** Widefield microscope image for GFP-Rad54, GFP-Rad54 S816A/S817A, and GFP-Rad54 S816D/S817D, and Rad54 D525S/D527S/S816D/S817D. **(H).** Mean fluorescent decay rate for Rad54 (Blue), Rad54 S816A/S817A (Green), Rad54 S816D/S817D (Orange), Rad54 D525S/D527S/S816D/S817D (Magenta) and Rad54 with ADP (Cyan). The black line gives the photobleaching rate. The shade represents the standard error measurement of the data. **(I).** Graph representing the *k*_*off*_ for GFP-Rad54 (+ATP), GFP-Rad54 S816A/S817A, and GFP-Rad54 S816D/S817D, Rad54 D525S/ D527S/S816D/S817D and GFP-Rad54 (+ADP) from dsDNA. The bars represent the mean, and the error bars the standard error measurement of the data.

Through our genetic analysis, we determined that substituting Aspartic acid for S816 and S817 disrupts D-loop stabilization *in vivo*. To better characterize the mechanism behind this defect, we purified Rad54, Rad54 S816A/S817A, Rad54 S816D/S817D, and Rad54 D525S/D527S/S816D/S817D (S10A Fig). A working hypothesis was that disruption of the interaction between S816/S817 and D525/D527 would impair communication between the two RecA lobes and may destabilize a specific conformation of Rad54. A logical consequence of this disruption may be a reduced affinity for dsDNA. Therefore, we initially tested the ability of these proteins to bind dsDNA using an Electrophoretic Mobility shift assay (EMSA) (Fig 5A). We quantified the apparent *K*_*d*_ and found a two-fold decrease in the affinity of Rad54 S816D/S817D mutant for dsDNA (**Fig 5B and 5C, 21 nM versus 41 nM, p = 0.008**). There was also a slight increase in the *K*_*d*_ for the Rad54 S817A/S817A mutant, but this change was not significant. Importantly, the *K*_*d*_ was restored to ~18 nM in the Rad54 D525S/D527S/S816D/S817D mutant. From this, we conclude there is a loss in the affinity for dsDNA in the Rad54 S816D/S817D mutant protein.

Rad54 is a dsDNA dependent ATPase, and a loss in affinity for dsDNA should result in a reduction in ATP hydrolysis. Therefore, we tested ATP hydrolysis for Rad54, Rad54 S816A/S817A, Rad54 S816D/S817D, and Rad54 D525S/D527S/S816D/S817D. As expected, the Rad54 S816D/S817D mutant hydrolyzed ATP with 10-fold lower efficiency than WT and the other two mutant versions of Rad54 (Fig 5D). We were surprised by the significant loss of ATP hydrolysis, but suspected that Rad54 S816D/S817D may still be activated in the presence of Rad51. The ATPase activity of Rad54 is stimulated by the presence of Rad51, leading to a 3–5-fold increase in ATP turnover [29,34,52]. We next tested whether Rad51 could still stimulate Rad54 S816D/S817D. Rad51 stimulated the mutant enzyme’s activity but did not restore it to WT levels (Fig 5E**, p = 0.01**). This stimulation is not due to an increase in Rad51 activity, whose ATP hydrolysis activity is at least 100-fold lower than Rad54. Previous reports have shown that there is no stimulation when Rad54 K341R, a catalytically inactive version of Rad54, is substituted in this experiment [52].

The defect in DNA binding was relatively small, and we sought to use a higher-resolution approach to directly measure Rad54’s dissociation rate. DNA curtains are a high-throughput single-molecule approach that can measure Rad54 dissociation rates (Fig 5F) [92]. In these experiments, GFP-Rad54 is injected into a flow cell and allowed to bind to dsDNA (Fig 5F and 5G). The loss of fluorescent signal is then measured over time in the presence of buffer flow. The rate of GFP photobleaching is also measured. If the protein dissociation rate is faster than the photobleaching rate, then a true dissociation rate of the protein can be determined (Fig 5H). We observed that the rate of dissociation for GFP-Rad54, GFP-Rad54 S816D/S817D, GFP-Rad54 S816A/S817A, and GFP-Rad54 D525S/D527S/S816D/S817D were all faster than photobleaching. By subtracting the bleaching rate from the fluorescent decay rate, we calculated the *k*_*off*_ for each protein (Fig 5I). We found that Rad54, Rad54 S816A/S817A, and Rad54 D525S/D527S/S816D/S817D all dissociated at ~1.3x10^-4^ sec^-1^, in contrast the Rad54 S816D/S817D mutant dissociated with a rate of 3.3x10^-4^ sec^-1^ (Fig 5I). This is a 2-fold difference and is consistent with the EMSA. Despite the small difference in dsDNA binding, the difference is significant (p < 0.0001), can be rescued by an intragenic suppressor, and is consistent across multiple techniques. From this, we conclude that Rad54 S816D/S817D is defective in binding dsDNA.

Rad54 can bind to DNA in the absence of ATP [93]. However, it has never been determined if there are differences in affinity for dsDNA during the ATP hydrolysis cycle. The AlphaFold model predicted that Rad54 underwent a conformational change that disrupted the D525-S816 interaction when bound to ADP versus ATP. From this, we generated a hypothesis that the ATP bound form of the enzyme has a higher affinity for dsDNA, and this conformation was disrupted in the S816D/S817D mutant. To test this, we measured the off-rate of GFP-Rad54 in the presence of ADP nucleotide. We found that the *k*_*off*_ was around 3-fold higher than the ATP-bound form (Fig 5I). From this, we conclude that the ADP bound form of the enzyme is less stable on dsDNA. This is consistent with a model where a loss of D525-S816 interaction fails to stabilize the ATP bound form of the enzyme in the S816D/S817D mutant.

Our data suggest that disruption of the Rad54 D525-S816 interaction may result in a loss of dsDNA binding and failure to form a D-loop. If this were true, then overexpression of the protein should result in complete rescue of the phenotype, because it would shift the binding equilibrium of Rad54 toward a bound state. Alternatively, failure to fully complement may suggest a more complex mechanism. To test this, we overexpressed *RAD54* and *rad54-S816D, S817D* from a 2-µ plasmid and assessed their ability to complement MMS sensitivity (S11A Fig). Overexpression of *rad54-S816D, S817D* did result in more efficient complementation than expression from a centromeric plasmid. However, it could not rescue to *RAD54* levels. We additionally evaluated survival using the ectopic repair assay. As with the MMS complementation, there was an increase in survival in ectopic recombination when *rad54-S816D, S817D* was expressed from a 2-µ plasmid (S11BC Fig**, 5% versus 16%, p = 0.03**). However, this did not complement the levels of *RAD54*. From this, we conclude that overexpression of the *rad54-S816D, S817D* cannot fully complement the phenotype, and that the mechanism by which it fails to promote D-loop formation is likely more complex.

### Rad54 S816D/S817D exhibits processivity defects in the absence of Rad51

Rad54 is a highly processive enzyme, meaning it can perform multiple ATP hydrolysis cycles before dissociating from or pausing on a dsDNA template. The energy released by ATP hydrolysis drives conformational changes in the Rad54 enzyme, and the final output is the translocation of the DNA. This mechanical activity can manifest as linear movement along the dsDNA or as extrusion of a DNA loop. Both translocation outcomes require Rad54’s processive activity. Recently, we determined that these two outputs can be controlled by the force applied to the DNA [94]. A simple way to measure Rad54’s processive activity is to monitor its distance and direction of movement along dsDNA using single-molecule imaging. In these experiments, we can add a nominal force from buffer flow to bias the translocation activity towards linear movement and away from loop extrusion.

**Fig 6:**
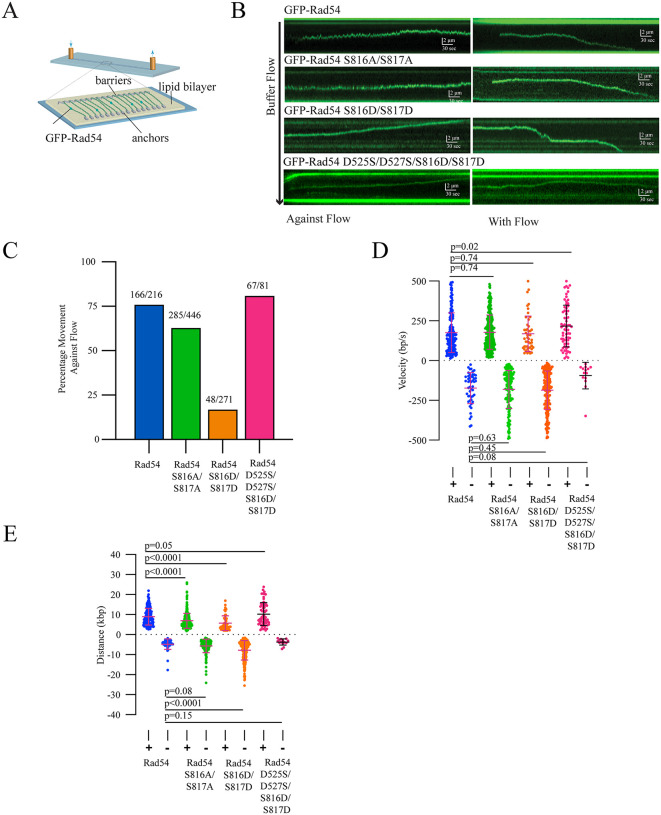
Rad54 S816D/S817D is poorly processive. **(A).** Schematic diagram illustrating DNA curtains experiments to test the activity of Rad54 on dsDNA. This image is reproduced through a CC-BY 4.0 license from [95]. **(B).** Representative kymographs illustrating the movement of GFP-Rad54, GFP-Rad54 S816A/S817A, GFP-Rad54 S816D/S817D, GFP-Rad54 D525S/D527S, S816D/S817D with and against buffer flow. **(C).** Bar graph representing the percentage of Rad54 (166/216), Rad54 S816A/S817A (285/446), and Rad54 S816D/S817D (48/271) and GFP-Rad54 D525S/D527S/S816D/S817D that move against the buffer flow. **(D).** Graph representing the velocity of translocation in kbp/s for Rad54 (N = 216), Rad54 S816A/S817A (N = 446), Rad54 S816D/S817D (N = 271), and Rad54 D525S/D527S/S816D/S817D (N = 81). The molecules that moved against the flow are above the X-axis, and the molecules that moved with the flow are below the X-axis. The bar represents the mean, and the error bars represent the standard deviation of the data. The negative value indicates the direction. **(E).** Graph representing the distance moved for Rad54 (N = 216), Rad54 S816A/S817A (N = 446), Rad54 S816D/S817D (N = 271), and Rad54 D525S/D527S/S816D/S817D (N = 81). The molecules that moved against the flow are above the X-axis (positive values), and the molecules that moved with the flow are below the X-axis (negative values). The bar represents the mean, and the error bars represent the standard deviation of the data.

We used DNA curtains to monitor the translocation activity of GFP-Rad54, GFP-Rad54 S816A/S817A, GFP-Rad54 S816D/S817D, and GFP-Rad54 D525S/D527S/S816D/S817D (Fig 6A). We divided the translocation tracks into those moving toward and away from the barriers (Fig 6B). Rad54 molecules moving against the flow will feel a slight opposing force, while those moving away from the barriers and with the flow will feel an assisting force. Under the conditions used here, a majority of Rad54 molecules (75%) moved toward the barriers. This is likely due to the DNA being more tense in that direction. The Rad54 S816A/S817A (63%) and the Rad54 D525S/D527S/S816D/S817D (82%) mutants also moved toward the barriers (Fig 6C). In contrast, only 17% of Rad54 S816D/S817D molecules moved toward the barrier. This is consistent with weaker-binding molecules being pushed down the DNA by the force of buffer flow.

Surprisingly, there was no difference in velocity (bp/sec) in either direction between any of the Rad54 variants tested, with the exception that there was a slight increase in the translocation rate of the Rad54 D525S/D527S/S816D/S817D mutant (Fig 6D**, p = 0.02**). Rad54 S816A/S817A and Rad54 S816D/S817D moved a significantly shorter distance against the flow than the WT (Fig 6E). This difference was rescued in the Rad54 D525S/D527S/S816D/S817D mutant (Fig 6E). The Rad54 S816D/S817D mutant moved farther along the DNA with the flow, consistent with a protein being pushed along the DNA. These data indicate that disruption of this interaction in Rad54 results in proteins that bind DNA less tightly, likely leading to lower processivity.

### PSCs with Rad54 S816D/S817D slip during translocation

**Fig 7:**
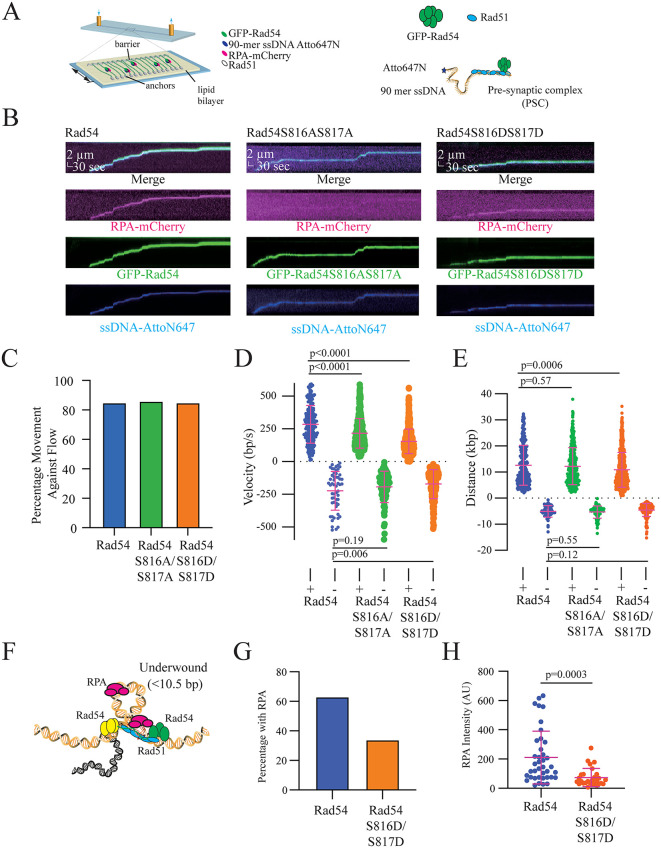
Mutations lead to PSC slippage during translocation. **(A).** Cartoon diagram illustrating the use of DNA curtains to perform experiments to monitor the activity of the presynaptic complex (PSC) on dsDNA. This image is reproduce through a CC-BY 4.0 license from [95]. **(B).** Representative kymographs for PSCs, PSCs with Rad54 S816A/S817A, or PSCs with Rad54 S816D/S817D. Shown are merged images of RPA-mCherry, GFP-Rad54, and Atto647–90-mer ssDNA. **(C).** Graph representing the percentage of PSC with Rad54 (183/215), PSC with Rad54 S816A/S817A (326/378), and PSC with Rad54 S816DS817D (710/835) that moved against the buffer flow. **(D).** Measured translocation velocities for the PSC with Rad54 (N = 215), PSC with Rad54 S816A/S817A (N = 378), and PSC with Rad54 S816D/S817D (N = 835). The PSCs that moved against the flow are above the X-axis, and the PSC values that moved with the flow are below the X-axis. The bars represent the mean, and the error bars represent the standard deviation of the data. **(E).** Measured translocation distances for the PSC with Rad54 (N = 215), PSC with Rad54 S816A/S817A (N = 378), and PSC with Rad54 S816D/S817D (N = 835). The PSCs that moved against the flow are above the X-axis, and the PSC values that moved with the flow are below the X-axis. The bars represent the mean, and the error bars represent the standard deviation of the data. **(F).** Schematic illustrating the loop extrusion activity of Rad54 extruding loops that can bind RPA-mCherry. **(G).** Graph representing the number of PSCs with RPA mCherry localized after a five-minute incubation without buffer flow for PSCs with Rad54 and Rad54 S816D/S817D. **(H).** A dot plot representing the intensity of RPA bound to bound to PSCs with Rad54 (N = 39) and Rad54 S816D/S817D (N = 28). The bar represents the mean of the data, and the error bars represent the standard deviation.

Rad51 is known to activate the ATP hydrolysis cycle of Rad54, thereby increasing translocation activity [29,95]. *In vitro*, Rad54 can bind to Rad51-ssDNA filaments, which can then bind to dsDNA and perform translocation [29,95]. This complex is referred to as the presynaptic complex (PSC) because it has not yet paired with a homologous DNA sequence (Fig 7A). We have previously used biochemical reconstitution of the PSC, combined with DNA curtains, to monitor Rad54 activity [29,95]. We next used DNA curtains to evaluate the impact of Rad51 on the linear translocation activity of Rad54 S816D/S817D.

As with the Rad54-alone experiments, these experiments were measured using flow-on to increase observation of linear movement during translocation. When experiments were performed with the PSC Rad54, Rad54 S816A/S817A, and Rad54 S816D/S817D, all three moved toward the barrier in ~80–85% of translocation events, marking the first difference in activity between the Rad54 S816D/S817D mutant alone and incorporated into the PSC (Fig 7B and 7C). In contrast to Rad54 alone, there was a difference in the translocation velocity between PSCs with Rad54, Rad54 S816A/S817A, and Rad54 S816D/S817D. The velocity of Rad54 S816A/S817A was 75% of WT (285 bp/sec versus 215 bp/sec, p < 0.0001, and Rad54 S816D/S817D was 54% that of WT (285 bp/sec versus 155 bp/sec, p < 0.0001). For the Rad54 S816A/S817A mutant, this difference was observed only during movement toward the barrier, with no difference in translocation events away from the barriers (Fig 7D). We observed no differences in translocation distance between WT and Rad54 S816A/S817A in either direction. There was a small but significant difference in the distance travelled between the WT and the Rad54 S816D/S817D PSC in the against-flow direction only (Fig 7E). Together, these data suggest that disruption of the Rad54 D525-S816 interaction reduces the rate of linear translocation. This likely stems from slippage of the Rad54 motor due to unstable binding to the dsDNA.

Our experiments suggested that Rad54 S816D/S817D PSC is defective in linear translocation. The PSC can also perform loop extrusion, generating stretches of underwound DNA. This DNA is competent to bind RPA, and the degree of RPA binding can report on the formation of an extruded loop. To establish competent loops that bind RPA, we performed experiments in which the PSC bound to DNA in the absence of buffer flow for 5 min. We then measured the percentage of PSCs that co-localized with mCherry-RPA and the intensity of the associated RPA (Fig 7F). Approximately 63% (53/83) of WT PSCs recruited RPA. In contrast, only 34% (47/138) of Rad54 S816D/S817D PSCs had RPA associated (Fig 7G). Additionally, the mean intensity of RPA bound to the PSC was only 35% of WT (Fig 7H). From this, we conclude that Rad54 S816D/S817D exhibits reduced loop extrusion activity and is defective in both translocation mechanisms.

From our previous work the consequences of loop extrusion are likely to be the recruitment of additional PSC molecules. An indicator of additional recruitment would be an increase in the amount of Rad54 present, and an increase in the amount of Rad51-ssDNA. This change would be evident by comparing the amount of Rad54 and ssDNA with and without buffer flow. By using a photobleaching standard, we directly measured the amount of GFP-Rad54 present with and without buffer flow. In WT, the number of GFP-Rad54 molecules increased two-fold when buffer flow was turned off to allow loop extrusion (S12A Fig**, p = 0.0007**). This observation was not true in the Rad54 S816D/S817D PSC, and there was an observed decrease in the number of Rad54 molecules when buffer flow was turned off (S12A Fig). It is important to note that WT and Rad54 S816D/S817D exhibit the same number of Rad54 molecules when buffer flow is left on (S12A Fig). A similar pattern was observed for fluorescently labelled ssDNA (S12B Fig). From this, we conclude that loop extrusion by Rad54 can recruit more PSC molecules, and this is significantly reduced when disrupting the D525-S816 interaction.

Finally, we measured the ability of the Rad54 S816D/S817D mutant protein to form D-loops i*n vitro.* We used a previously established assay to show that while Rad54 could promote recombination on a supercoiled plasmid, Rad54 S816D/S817D was unable to promote D-loop formation (S12C Fig). From this we conclude that Rad54 S816D/S817D is defective for D-loop formation both *in vitro* and *in vivo*.

## Discussion

In this study, we identified an intra-protein interaction in Rad54. The disruption of this interaction reduces enzyme function by destabilizing contact between the two RecA lobes and reduces binding to dsDNA. The consequence of this disruption is a protein that fails to form D-loops both *in vivo* and *in vitro*. However, it remains active in removing Rad51 that is pathologically bound to dsDNA. It can also complement *rad54∆* strains in the presence of certain DNA-damaging agents, which could be a result of reduced function or separation of function. Our data indicate that the Rad54 mutant slips during DNA translocation, leading to severely diminishing its capacity to catalyze D-loop formation.

Brownian ratchets are molecular machines that harness thermal fluctuations in proteins to perform useful directional work. This action is usually achieved through binding a cofactor, such as ATP, which traps proteins in defined conformations. Rad54 can be considered a Brownian ratchet with ATP hydrolysis guiding the protein through a series of conformational changes. A general hypothesis from our data is that the interaction between D525 and S816 is required to stabilize a specific Rad54 conformation during the ATP hydrolysis cycle and the resulting translocation event. Disruption of this interaction leads to defects in the enzyme’s processivity.

**Fig 8:**
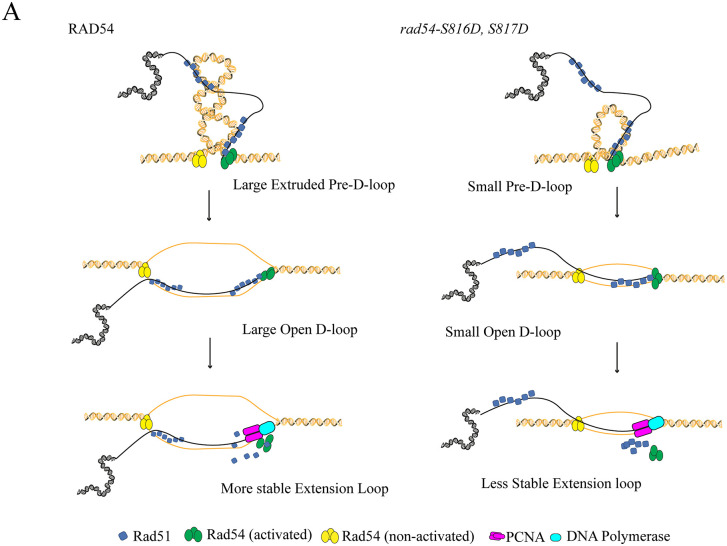
Loop extrusion mechanism for D-loop formation. A cartoon diagram illustrating a proposed mechanism for Rad54 mediated D-loop formation, and the consequences of *rad54-S816D, S817D* failure during the process. We propose that Rad54 promotes homology search by extruding DNA loops and locally pumping duplex DNA to facilitate Rad51-catalyzed strand invasion, leading to the formation of a D-loop intermediate. Following strand invasion, Rad54 removes Rad51 from the ssDNA 3’end, allowing DNA polymerase-mediated extension that stabilizes the D-loop. In the *rad54-S816D, S817D* mutant, reduced DNA binding affinity and processivity impair loop extrusion, resulting in smaller or less stable pre-D-loop intermediates and reduced efficiency of D-loop formation and extension.

Our single-molecule experiments illustrated different aspects of this model. For example, Rad54 alone hydrolyzes ATP more slowly than when in complex with Rad51. A slow rate of ATP hydrolysis will result in increased dwell times of specific Rad54 conformations, leading to a greater requirement of the D525-S816 interaction to remain bound to the DNA. This manifests as dissociation from the dsDNA template or being pushed along the DNA by the buffer flow. A difference in translocation velocity would likely be seen in the S816D/S817D mutant as well, but the motor’s instability prevents this population from being observed. When Rad51 is present, the ATP hydrolysis cycle is shorter. Under these conditions, Rad54 is likely to pass through the stabilized conformation more rapidly, thereby reducing its lifetime in this state. Therefore, disruption of the D525-S816 interaction only causes slippage of the enzyme, and not dissociation. Loss of these interactions then reduces the apparent velocity along the DNA and loop extrusion activity. Importantly, in the Rad54 S816D/S817D, we see a reduction in RPA recruitment and the number of PSC molecules that localize with Rad54 under conditions that favor loop extrusion. These data argue that a pre-D-loop or homology search complex is significantly smaller, and does not form efficiently (Fig 8).

Importantly, Rad54 slippage has critical physiological implications for D-loop formation. Our data suggest that disruption of this interaction does not affect Rad54’s ability to remove Rad51 from dsDNA. It should be noted that our measure of this activity is indirect and does not occur during double strand break repair. Still, the assay used here has been an essential assay in developing the model that removal of Rad51 from a newly paired D-loop is the mechanism of D-loop stabilization [37]. It remains an open question how processive Rad54 must be to remove Rad51 from dsDNA. Single molecule experiments suggest that filaments of Rad51 can be disrupted by eliminating the 3’ ATP bound Rad51 promoter, leading to filament collapse due to tension on the donor dsDNA [96]. If the disruption of Rad51 filaments were the mechanism of D-loop stabilization, then this action could occur without processive activity. Importantly, removal of Rad51 from dsDNA is an important activity but may occur after stable D-loop formation to promote DNA extension [50].

An alternate model for D-loop formation that requires processive action of Rad54 is the extrusion and stabilization of a topologically isolated DNA loop, as previously proposed [47]. Stabilization of an extruded loop by processive Rad54 activity may render DNA in the dsDNA in the loop prone to strand separation by melting during Rad51-mediated strand exchange (Fig 8). The size of this extruded D-loop may ultimately impact the overall stability of the primary D-loop by promoting greater base pairing potential. This model would make D-loop formation prone to failure if Rad54’s processive activity is unstable or if the loops rapidly dissociate. The apparent 2-fold defect in the Rad54 S816D/S817D mutant is minor. However, this defect will be amplified if there is a 2-fold increase in the chance of loop failure for each successive ATP hydrolysis event. Current models of chromatin remodeling enzymes predict that a single base pair is translocated per ATP hydrolysis event [97,98]. If this is also the case for Rad54, the probability of slippage is amplified over the length of the translocation event. If this event requires 90–100 bp of processive action to form a D-loop, then a 2-fold defect will increase the failure of stabilization over each of the 100 steps needed to extrude and stabilize the loop.

### Phosphorylation at S816 is likely a repressive mark

Phosphorylation of S816 was identified in two independent phosphoproteomic screens [69,70]. SILAC experiments suggested phosphorylation may occur under damaging conditions during the G1 phase of the cell cycle. The specific kinase is unclear. Based on our experiments, S816 phosphorylation is likely a repressive mark that downregulates Rad54 activity. Inactivation would help reduce recombination during G1, when the sister chromatid is absent. The most likely scenario is phosphorylation during G1 to reduce the potential for ectopic recombination during repair of double-strand breaks, increasing the fidelity of the genome throughout the cell cycle. Future work should be designed to identify the specific kinase responsible for phosphorylating Rad54.

### Interactions with Sgs1

We identified an unexpected genetic interaction between *RAD54* and *SGS1*. The specific details of this interaction are still unclear. We observed in the *rad54-S816A, S817A sgs1∆* strain that there was a suppression of the increased amount of second-strand DNA synthesis observed in the *sgs1∆* strain using a BIR-like system. A reasonable explanation is that in the *rad54-S81A, S817A sgs1∆*, there is a delay in the transition from strand pairing to the next phase in DNA repair synthesis, resembling the WT. We did not observe a reduction in the level of ssDNA extension. However, we did not perform a detailed kinetic analysis of this process, and a delay in timing is possible. The delay caused by the *rad54-S816A, S817A* allele may compensate for the loss of *SGS1* and reduce the transition to a migrating bubble, lowering the efficiency of the second-strand synthesis on the newly synthesized ssDNA. This hypothesis is highly speculative, and further testing will be required to confirm it.

The interaction observed between *rad54-S816D, S817D*, and *SGS1* is significantly more complicated to interpret. We observed a decrease in short tract gene conversion, and a decrease in extension from nascent D-loops. One possibility is that the D-loops formed in the *rad54-S816D, S817D* strain are short and structurally distinct from those formed in WT. This difference may alter Sgs1’s role or prevent its proper function. Alternatively, *SGS1* is involved in DNA end-resection and the dissolution of dHJ intermediates [75,99,100]. Deletion of *SGS1* delays the kinetics of end resection [6,101], potentially disrupting the overall timing of the recombination reaction. Similarly, Rad54’s failure to efficiently stimulate strand exchange may impose similar kinetic limitations on the recombination reaction. Therefore, the combined disruptive effects of *RAD54* and *SGS1* on the recombination reaction manifest as reduced DNA extension, altering gene conversion, and chromosome loss as the probability of a successful strand exchange is reduced.

## Conclusions

In this study, we identified a conserved mutation in Rad54 that reduces binding to dsDNA. This amino acid substitution caused a severe defect in D-loop formation *in vivo* but did not alter the protein’s ability to remove Rad51 from double-stranded DNA *in vivo*. From this, we hypothesize that this is a separation of function mutant. This apparent separation of function enables us to better understand Rad54’s sequential action at initial strand-exchange intermediates, thereby unifying two existing models in the field. Future work should focus on understanding how Rad54’s loss of DNA-binding affects outcomes in human cell lines and at DNA replication forks.

### Limitations of this study

A major limitation of this study is that we do not use physical methods to directly detect an interaction that may occur between the two RecA lobes of Rad54. Future work should focus on resolving the physical relationship between the two RecA lobes of Rad54. Ideally, this will come on the form of a high-resolution structure.

## Materials and methods

### Yeast strains construction

Strains for the initial spot assay were BY4741 and generated by centromeric plasmid expression in the *rad54∆* strain or by gene replacement, as indicated in the context. Strains for the ectopic recombination assay were TGI354 [75] and were generated by gene replacement or centromeric plasmid expression in the *rad54∆* strain, as indicated in the context. Strains for the *in vivo* D-loop capture (DLC) and extension (DLE) assay were generated in the WDHY5511 [19,80] background by gene replacement. Strains for red/white allelic recombination were generated by gene replacement from parent strains LSY2205 and LSY2202 [87]. Strains in W303, TGI354, and WDHY5511 backgrounds were also used in the spot assay as indicated. All yeast strains used in this study are listed in S1 Table and all plasmids are listed in S2 Table.

### Protein purification

Rad54, Rad54 S816A/S817A, Rad54 S816D/S817D, Rad51, and RPA-mCherry were purified as previously described [29]. In brief, a protease-deficient yeast strain was transformed with GFP-GST-Rad54, GFP-GST-Rad54 S816A/S817A, or GFP-GST-Rad54 S816D/S817D on a 2-µ plasmid under the control of the Gal1/10 promoter. Cells were grown in YNB (–Ura) plus 3% glycerol and 2% lactic acid. When the cells reached an OD_600_ of 1.5, expression was induced by adding 2% galactose for 6 hours. Cells were harvested by centrifugation and stored at –80 °C.

Cell pellets were resuspended in Rad54 resuspension buffer (30 mM Tris–Cl [pH 7.5], 1 M NaCl, 1 mM EDTA, 10% glycerol, 10 mM BME (β-mercaptoethanol), protease inhibitor cocktail and 2 mM PMSF). Cells were disrupted by manual bead beating, and the lysate was clarified by centrifugation at 26,500 x g for 1 hour. The lysate was fractionated by ammonium sulfate (AS) precipitation. AS was gradually added with mixing to a final concentration of 20% followed by centrifugation at 10,000 x g for 10 minutes. The supernatant was discarded, and the AS concentration was raised to 50% followed by centrifugation at 10,000 x g for 10 min. The protein pellet was resuspended in PBS (phosphate buffered saline) plus 1M NaCl and 10 mM BME. The resulting re-suspended protein was then bound to pre-equilibrated GST resin in batch for 1 hour at 4°C. The GST resin was washed twice with PBS plus 1000 mM NaCl, and twice with PBS plus 500 mM NaCl. The protein was eluted in 20 mM glutathione in PBS plus 500 mM NaCl. The peak fractions were pooled and then applied to a Sephacryl S–300 High Resolution gel filtration column pre-equilibrated with Rad54 SEC buffer (30 mM Tris–Cl [pH 7.5], 500 mM NaCl, 1 mM EDTA, 10% glycerol, and 10 mM BME). The peak was pooled and dialyzed against Rad54 SEC buffer plus 50% glycerol and stored at –80 °C in single-use aliquots.

6xHis-SUMO-Rad51 was transformed into *Escherichia coli* BL21 (DE3) Rosetta2 cells and grown to an OD_600_ of 0.4 to 0.6 at 37 °C. Expression was induced by addition of 0.5 mM IPTG for 3 hours at 37°C. Cells were harvested and stored at –80 °C. Cells were lysed by freeze–thaw in Cell Lysis Buffer (CLB: 30 mM Tris–Cl [pH 8.0], 1 M NaCl, 10% glycerol, 10 mM imidazole, 5 mM BME, and protease inhibitor cocktail). Crude lysates were sonicated for 6 pulses of 30 seconds on and 2 minutes off, then clarified by centrifugation at 26,500 x g. The extract was precipitated with 50% AS and centrifuged at 26,500 x g for 10 minutes. The pellet was resuspended in CLB and bound to 1 mL of pre–equilibrated Ni–NTA resin for 1 hour with rotation at 4 °C. The resin was washed three times with CLB and eluted in CLB plus 200 mM imidazole. The protein was mixed with 400 units of the SUMO protease Ulp1 and dialyzed overnight at 4 °C into Rad51 buffer (30 mM Tris–Cl [pH 8.0], 150 mM NaCl, 1 mM EDTA, 10% Glycerol, 10 mM imidazole). The 6xHis–SUMO tag and SUMO protease were removed by passing the dialyzed proteins over a second 1 mL Ni–NTA column. The purified Rad51 was then stored at –80 °C in single-use aliquots.

### Multiple sequence alignment

Rad54-like eukaryotic sequences were retrieved by BLASTP (NCBI, nr database; organism filter: Eukaryota) using *S.c.*Rad54 as the query. Redundant entries and obvious fragments were removed. Sequences were aligned with Clustal Omega, the alignment was trimmed with trimAl, and sequence logos were generated from the trimmed alignment using the Python package Logomaker.

### Yeast spot growth assay

*RAD54* and its designed mutants were expressed using a centromere vector, pRS415, and transformed into the BY4741 *rad54* strain or other background strains as indicated, followed by selection on YNB (–Leu) + 2% dextrose plates. Transformed cells were grown overnight in YNB (–Leu) + 2% dextrose medium. The following day, the overnight cultures were diluted to an OD_600_ of 0.3 and grown to an OD_600_ of 1.0. Cells were then serially diluted and spotted on YNB (–Leu) + 2% dextrose plates containing either no drug, 0.01% methyl methanesulfonate (MMS), or 0.02% MMS. Other drugs were also used, including 20 mM Hydroxyurea, 0.02 µg/mL 4-NQO, 0.04 µg/mL 4-NQO, 10 µM camptothecin (CPT), and 20 µM CPT as indicated. Plates were incubated at 30 °C for 3 days and imaged at 72 hours. BY4741 strains with *RAD54* mutants were also generated via gene replacement. The procedures for the spot assay for these strains were the same as for the centromeric-expressed strains, except that the overnight culture was grown and diluted in Yeast extract + Peptone (YP) + 2% dextrose medium. The serially diluted cells were spotted on YP + 2% dextrose containing either no drug or the drugs mentioned above. Plates were incubated at 30 °C for 2 days and imaged at 48 hours. For *RAD51* or *rad51I345T* on a pYES plasmid, cells were transformed. Cultures were grown overnight in YNB (–Ura) + 2% dextrose and diluted the next day. After cells reached an OD_600_ of 1.0, they were serially diluted onto YNB (–Ura) + 2% dextrose or YNB (–Ura) + 2% galactose and incubated at 30 °C for 48 hours. They were then images.

### Ectopic recombination assay

The wildtype strain used for this study was described in [75]. The genotypes for modifying these strains are listed in S1 Table. A single colony was picked and grown in YP + 3% glycerol + 2% lactate medium to log phase. The culture was diluted and plated on YP + 2% dextrose and YP + 2% galactose plates, respectively. Plates were incubated at 30 °C for 2–3 days, and the number of colonies was counted. The viability rate was calculated by dividing the colony number on the YP + 2% galactose plate by that on the YP + 2% dextrose plate. The mean and standard deviation were calculated for multiple independent experiments as indicated. For centromeric expression of RAD54 and its mutants in *rad54* strains, a single colony was picked and grown in YNB (–Leu) + 2% dextrose overnight. On the second day, the culture was diluted 10-fold into YNB (–Leu) containing 3% glycerol and 2% lactate and grown at 30 °C for 6 hours. Then the culture was diluted and plated onto YNB (–Leu) + 2% dextrose and YNB (–Leu) + 2% galactose, respectively. After being incubated at 30 °C for 3–4 days, the numbers of colonies were determined, and the viability was calculated by dividing the colony number on the galactose plate by that on the dextrose plate.

### Allelic Recombination between homologs assay

The assay was performed by growing the appropriate strain overnight in YP + 2% raffinose. The next day, cells were diluted to an OD_600_ of 0.2 and allowed to reach an OD_600_ of 0.4-0.5, then *I-Sce1* expression was induced by adding 2% galactose. Cells were allowed to grow for an additional 1.5 hours, then plated onto YP + 2% dextrose (YPD) plates and grown for 48 hours. After 48 hours, they were placed at 4 °C overnight to enable further development of the red color. The number of white, red, and sectored colonies was then counted, followed by replica plating onto YPD + hygromycin B (200 µg/ml) and YPD + nourseothricin (67 µg/ml, clonNat) for analysis of recombination outcomes. Strains were also replica plated on YNB (–Ura or –Met) + 2% dextrose to ensure proper chromosome segregation and YNB (–adenine sulfate) + 2% raffinose +1% galactose to assay for recombinants versus uncut DNA. Finally, colonies were replica plated on YNB (–adenine sulfate) + 2% dextrose to directly count short tract gene conversion events. The data were analyzed by counting sectored colonies and assessing colony survival across different antibiotic sensitivities. The data for each category was then divided by the total population of sectored colonies.

### Yeast cell imaging

To immobilize yeast cells, an agarose pad was used. A 1% agarose solution was prepared by adding agarose in S media (YNB + 0.5% ammonium sulfate, then autoclaved) and microwaving to dissolve. An amino acid mix was added to a final concentration of 1x to ensure yeast cells could grow on the agarose pad. The dissolved agarose was kept warm on a 65 °C metal heater. One clean microscope slide was taped at both ends, and 30 µL of 1% agarose was applied to the center of the slide. Another clean and untapped microscope slide was immediately placed on top, and gentle pressure was applied to ensure the agarose spread evenly. Then the slide sandwich was placed on an ice bag for approximately 30 seconds to allow the agarose to solidify. The top slide was carefully slid out, leaving the agarose pad intact on the bottom slide. The agarose pad was allowed to dry for 3 minutes before 3 µL of properly diluted yeast cells was added. A coverslip was then placed over the cells. A Nikon Eclipse Ti microscope equipped with a 488-nm laser. A Plan APO 60XA/1.20 WI objective and an ANDOR Zyla-5.5-CL3 camera were used to observe and capture yeast cells.

### ATPase assay

A commercially available ADP-GLO kit (Cat No. V6930, Promega) was used to measure ATP hydrolysis activity. ATP hydrolysis reaction was performed in HR buffer (20 mM Tris-OAc [pH 7.5], 50 mM NaCl, 10 mM Mg(OAc)_2_, 200 ng/μl BSA, 1 mM DTT, and 10% Glycerol) and contained 1 mg/ml sheared salmon sperm DNA, 20 nM Rad54, and 200 nM Rad51 (if added).

### Electrophoresis mobility shift assay (EMSA) for Rad54

An Atto647N-labeled 90-mer oligo was annealed with an unlabeled complementary oligo to form a labeled 90-bp dsDNA substrate. The oligo sequences are available in S3 Table. The binding reaction was performed in EMSA buffer (35 mM Tris-Cl [pH 7.5], 3 mM MgCl_2_, 50 mM KCl, 1 mM DTT, 10% glycerol). The final DNA concentration was 10 nM, and proteins were titrated to be 0, 6.25, 12.5, 25, 50, 100, 150, and 200 nM as final concentrations for Rad54 wildtype and Rad54 S816D/S817D. Rad54 S816A/S817A was titrated to be 0, 6.25, 12.5, 25, 50, 100, and 139 nM as final concentrations. The DNA and proteins were incubated at 30 °C for 5 min and then resolved by 8% Native-PAGE in 0.5x TBE buffer (44.6 mM Tris, 44.5 mM boric acid, 1 mM EDTA, 8% acrylamide/bis-acrylamide (37.5:1), 0.1% APS, 0.1% TEMED) running in 0.5x TBE buffer (44.6 mM Tris, 44.5 mM boric acid, 1 mM EDTA). The hill curve of Rad54-DNA binding was fitted through GraphPad prism.

### *In vitro* D-loop assay

D-loop formation experiments were performed in HR buffer (30 mM Tris-OAc [pH 7.5], 50 mM NaCl, 10 mM Mg(OAc)_2_, 1 mM DTT, 0.2 mg/ml BSA) using an Atto647N-labeled DNA duplex (15 nM) with homology to the pUC19 plasmid. Rad51 (300 nM) was incubated with recipient DNA at 30 °C for 15 minutes. The resulting Rad51 filaments were mixed with indicated concentrations of Rad54, RPA (500 nM), and pUC19 plasmid (0.3 nM). Reactions were quenched at the indicated time points and treated with 1 unit of Proteinase K at 37 °C for 20 minutes. The reactions were then resolved by electrophoresis on a 0.9% agarose gel and imaged for fluorescence using a Typhoon imager.

### Flow cell construction

Metallic chrome patterns were deposited on quartz microscope slides with predrilled holes for microfluidic line attachment by electron beam lithography to generate flow cells. After metal deposition, a channel was created by placing a small piece of paper between the drill holes and covering the two-sided tape. The paper was excised to make the flow chamber, and a glass coverslip was fixed to the tape. The chamber was sealed by heating to 165 °C in a vacuum oven at 25 mmHg for 60 min. Flow cells were then completed by hot-gluing IDEX nano ports over the drill holes on the opposite side of the microscope slide from the coverslip.

### Single molecule experiments

All single molecule experiments were conducted on a custom-built prism-based total internal reflection microscope (Nikon) equipped with a 488-nm laser (Coherent Sapphire, 100 mW), a 561-nm laser (Coherent Sapphire, 100 mW), a 640-nm laser (Coherent Obis, 100 mW) and two Andor iXon EMCCD cameras. DNA substrates for DNA curtains experiments were made by attaching a biotinylated oligo to one end of the 50 kb Lambda phage genome and an oligo with a digoxigenin moiety on the other. This enabled double tethering of the DNA between the chrome barriers and the chrome pedestals, as previously described. As for single tethered DNA, if specified in the context, flow cells were attached to a microfluidic system, and sample delivery was controlled using a syringe pump (KD Scientific). Three-color imaging was achieved by two XION 512 × 512 back-thinned Andor EM-CCD cameras and alternative illumination using a 488 nm laser, a 561 nm laser, and a 640 nm laser at 25% power output. The lasers were shuttered, resulting in a 200-msec delay between each frame. Images were collected with a 200-msec integration time. Translocation velocity and distances were measured in HR Buffer. Channel bleed-through is prevented by shuttering of the laser lines, emission filters, and the use of complementary fluorophores. In this case GFP is not activated by the 561 or 647 laser lines, and the 488 or 561 laser lines do not activate Atto647N. mCherry labelled the 488-laser line can poorly activate fluorophores. However, the emitted light is split by a dichroic mirror and filtered through a band-pass and long-pass filter to block wavelengths of light above a certain cutoff. This is sufficient to prevent mCherry signal bleed through into the 488 channel.

### Analysis of dsDNA translocation

The velocity and track length for GFP-Rad54 molecules were measured by importing raw TIFF images as image stacks into ImageJ. Kymographs were generated by pointing around a fluorophore signal and defining the whole individual dsDNA molecules where the fluorophore bound to a region of interest (ROI) using a home-made script. Data analysis was performed from the kymographs. The start of translocation was defined when the GFP-Rad54 molecule moved > 2 pixels. Pauses were defined as momentary stalls in translocation that lasted 2–4 frames. Termination was defined by molecules that did not move for > 10 frames. Velocities were calculated using the following formula [(Y_f_–Y_i_) ×1031.96 bp/ [|X_f_–X_i_|]) ×frame rate]; where Y_i_ and Y_f_ correspond to the initial and final pixel position and X_i_ and X_f_ correspond to the start and stop time (in seconds). Negative values stand for translocations moving from barrier to pedestals, which was assisted by a flow. Positive values stand for translocations against flow directions.

### Dissociation rate measurement

The dissociation rate of DNA-GFP-Rad54 was measured by initiating data collection, followed by the injection of 10nM GFP-Rad54 in HR buffer onto single-tethered DNA curtains to allow proteins to bind to DNA. The flow rate was maintained at 0.2 mL/min to allow for initial binding and prevent further binding events. Images were collected with a 100 msec integration time at 200 msec intervals for a duration of 5 minutes for the bleaching experiment, or at 1 sec intervals for a period of 15 minutes for the dissociation experiment. The fluorescent signal decay in both experiments was fit to an exponential function, $A=A_{max}e^{-kt}$. The difference between the k values obtained from bleaching experiment and dissociation experiment was used to calculate the dissociation rate:$k_{off}=k_{dissociation}-k_{bleaching}$.

### DLC assay

DLC assay was performed as described before [19,80]. In brief, yeast cells were grown in a 5 mL YP + 2% dextrose + 4% adenine sulfate medium overnight. The second day, the culture was diluted by 10-fold in 5 mL YP + 3% glycerol + 2% lactate + 4% adenine sulfate and grown for around 8 hours. Then the culture was inoculated into 100 mL of YP + 3% glycerol + 2% lactate + 4% adenine sulfate medium with an initial OD_600_ ≈ 0.006 and grown for 16 hours. A 5x psoralen stock solution (0.5 mg/mL trioxsalen in 200-proof ethanol) was made in a 50-mL aluminum foil-covered falcon tube and dissolved on a shaker at room temperature overnight with gentle rocking. Next day, the culture should have an OD_600_ at 0.3-0.8. 7.5 OD_600_ of cells was collected as time 0 control, centrifuged at 2,246 x g, 4 °C for 5 minutes.

The cell pellets were resuspended in 1x psoralen buffer. The 1x psoralen buffer was prepared by diluting 5x psoralen in 200-proof ethanol before collecting cells. The resuspended cells were plated in a 60 mm x 15 mm petri dish, put 2–3 cm below a UV light source with the lip removed atop a pre-chilled metal block. The cell samples were exposed under the UV light for 10 minutes with gentle shaking to crosslink DNA. The cells were transferred to a 15-mL Falcon tube. The petri dish was rinsed with TE1 solution (50 mM Tris-Cl [pH 8.0], 50 mM EDTA), and the TE1 buffer was poured over the cells. The cells were then centrifuged at 2,246 x g, 4 °C for 5 minutes again. The supernatant was properly disposed of, and the pellets were stored at –20 °C. Galactose was added to the culture to a final concentration of 2% to induce DSBs. Cells were collected at the designed time points as described above. For lysis, the cell pellets were thawed on ice, then resuspended in spheroplasting buffer (0.4 M sorbitol, 0.4 M KCl, 40 mM sodium phosphate buffer [pH 7.2], 0.5 mM MgCl_2_) and transferred to a 1.7 mL microfuge tube.

The cells were spheroplasted in zymolyase solution (2% glucose, 50 mM Tris-Cl [pH 7.5], 5 mg/mL zymolyase 100T) at 30 °C for 20 minutes. The cells were washed with spheroplasting buffer three times at 2,500 x g and restriction enzyme buffer (RE buffer: 50 mM potassium acetate, 20 mM Tris-OAc, 10 mM Mg(OAc)_2_, 1 mg/mL BSA) at 16,000 x g three times. The pellets were resuspended in 1.4x RE buffer, either alone or with a hybridization oligo to restore the *EcoR*I restriction sites, and stored at –80 °C. The DNA was solubilized by incubating the cells with 0.1% SDS at 65 °C for 13 minutes. 1% Triton X-100 quenched the SDS. The DNA was digested by 20 U *Eco*RI at 37 °C for 1 hour. The restriction enzyme was deactivated by incubating the DNA with 1.5% SDS at 55 °C for 10 minutes. The cells were returned to ice, and SDS was quenched by the addition of 6% Triton X-100. Ligation buffer (50 mM Tris-Cl [pH 8.0], 10 mM MgCl_2_, 10 mM DTT, 2.5 μg/mL BSA, 1 mM ATP, pH 8.0, 8 U T4 DNA ligase) was added to perform the ligation reaction at 16 °C for 1 hour and 30 minutes. 25 μg/mL protease K was added to digest the enzymes at 65 °C for 30 minutes. DNA was extracted by adding phenol:chloroform:isoamyl alcohol and vortexing. The upper water phase was moved and incubated with a tenth volume of sodium acetate and a volume of isopropanol at room temperature for 30 minutes and centrifuged at 21,130 x g, 4 °C for 10 minutes to get DNA precipitation. The DNA pellets were dried at 37 °C and dissolved by incubating with 1xTE buffer (10 mM Tris-Cl [pH 8.0], 1 mM EDTA) at 37 °C for 1 hour. The DNA was used as a qPCR template with the primers listed in S3 Table. DLC chimera content was calculated by [DLC amplification efficiency]^[-Cp_(DLC)_], and the intramolecular ligation product content was calculated by [intramolecular ligation amplification efficiency]^[-Cp_(ligation)_]. The final DLC signal was calculated as DLC chimera content divided by intramolecular ligation product content.

### DLE assay

DLE assay was performed as described before [19,80]. The procedures were similar with DLC assay except for (1) 2.5 OD_600_ of cells were collect at each time point; (2) DNA crosslinking was omitted and cell pellets were washed by TE1 buffer twice at 2,246 x g; (3) hybridization oligos were different and listed in S3 Table; (4) DNA was solubilized by 1% SDS at 65 °C for 15 minutes; (4) DNA digestion was performed by adding 20 U *Hin*dIII; (5) qPCR was performed using some different oligos listed in S3 Table.

## Supporting information

S1 Table Strains used in this study. (PDF)

S2 Table Plasmids used in this study. (PDF)

S3 Table Oligos Used in this study. (PDF)

S1 Fig *rad54-S816D*, *S817D* is more deficient than *rad54-S816D.* (A). Serial dilution spot assay to determine the sensitivity of yeast strains with single amino acid substitutions at *Rad54 S816* and *S817* or double mutations at the same positions. Serine was mutated to either Alanine or Aspartic acid.
(TIF)

S2 Fig Mutant forms of Rad54 are expressed in cells. (A). Representative western blot illustrating the expression of *RAD54-GFP*, *rad54-S816A, S817A-GFP*, and *rad54-S816D, S817D-GFP* with and without 0.01% MMS. PGK1 is used as a loading control. The Asterix represents a non-specific band. (B). Fluorescent microscope images illustrating that *RAD54-GFP*, *rad54-S816A, S817A-GFP*, and *rad54-S816D, S817D-GFP* form foci in response to MMS treatment. (C). Bar graph quantifying the percentage of cells that form foci in response to MMS treatment.
(TIF)

S3 Fig Computational analysis of potential phosphorylation sites on Rad54. (A). AlphaFold3 model for *S. cerevisiae* Rad54 bound to dsDNA, colored for confidence with the pLDDT formation. Structures predicted with ATP and ADP are overlayed. (B). AlphaFold predictions for Rad54 without DNA and with ATP (Top, Left), Rad54 without DNA with ADP (Top, Right), AlphaFold predictions for Rad54 with DNA and with ATP (Bottom, Left), Rad54 with DNA with ADP (Bottom, Right). Residues D525, D527, S816, and S817 are highlighted. All structures are color coded with pLDDT colors as listed in the figure legend in A.
(TIF)

S4 Fig *rad54-D525A* and *rad54-D525K* phenocopy *rad54-S816D*, *S817D* mutants. (A). Yeast spot assays testing the ability of *rad54-D525A* and *rad54-D525K* to complement 4-NQO, CPT, and HU sensitivity.
(TIF)

S5 Fig Excerpt from multiple sequence alignment. (A). An excerpt from the Rad54 multiple sequence alignment. Shown are the two interacting regions on lobes 1 and 2, respectively. Included in this alignment in *Dictyostelium discoideum,* which has a naturally occurring Aspartic acid in place of Serine on lobe 2.
(TIF)

S6 FigSupplementary information for the DLC assay.(A). Chromosome schematic for the DLC assay. (B). Workflow for the DLC assay. (C). DLC signal at 4 hours. Column set 1 shows results with a hybrid oligo added; column set 2 shows results without adding a hybrid oligo. (D). DNA loading control (Cp values for amplicons at *ARG4*) for *RAD54; rad54∆; rad54-S816A, S817A; rad54-S816D, S817D sgs1∆; rad54-S816A, S817A sgs1∆; rad54-S816D, S817D sgs1∆; and rad54-D525S, D527S, S816D, S817D.* Columns: (1) 4 hours with hybrid oligo; (2) 4 hours without hybrid oligo. (E). Intramolecular ligation efficiency calculated as $\frac{Intramolecularligationamplificationefficiency^{-Cp_{\left(ligation\right)}}}{ARG\text{4}amplificationefficiency^{-Cp_{\left(ARG\text{4}\right)}}}$. Columns as in (D). (F). Crosslinking efficiency calculated as $\frac{ssDNAamplificationefficiency^{-Cp_{\left(ssDNA\right)}}}{ARG\text{4}amplificationefficiency^{-Cp_{\left(ARG\text{4}\right)}}}$. Columns as in (D). (G). DNA content at HO cut sites,calculated as $\frac{HOcutsiteamplificationeffieciency^{-Cp_{\left(HOcs\right)}}}{ARG\text{4}amplificationefficiency^{-Cp_{\left(ARG\text{4}\right)}}}$. The “0h” value represents the mean across all available 0-hour time point groups from all genotypes with a hybrid oligo added. Other columns represent DNA content at HO cut sites at 4-hour timepoint.(TIF)

S7 FigSupplementary information for the DLE assay.(A). Chromosome schematic for the DLE assay. (B). Workflow for the DLE assay. (C). DNA loading control (Cp values for amplicons at *ARG4*). For *RAD54; rad54∆; rad54-S816A, S817A; rad54-S816D, S817D; sgs1∆; rad54-S816A, S817A sgs1∆; rad54-S816D, S817D sgs1∆; and rad54-D525S, D527S, S816D, S817D.* (D). Intramolecular ligation efficiency calculated as $\frac{Intramolecularligationamplificationefficiency^{-Cp_{\left(ligation\right)}}}{ARG\text{4}amplificationefficiency^{-Cp_{\left(ARG\text{4}\right)}}}$ for allgenotypes. (E). DNA content at HO cut sites $\frac{HOcutsiteamplificationeffieciency^{-Cp_{\left(HOcs\right)}}}{ARG\text{4}amplificationefficiency^{-Cp_{\left(ARG\text{4}\right)}}}$ for all genotypes at 0-hour and 6-hour time points with hybrid oligos added. Numbers above the bars indicate the reduction in DNA content at the HO cut site after 6 hours. (F). Extension products at 6 hours: single-stranded (ssDNA; DLE signal when both hybrid oligos were added) and double-stranded (dsDNA; DLE signal when no hybrid oligos were added) for all genotypes. The error bars represent the standard error measurement of at least three independent experiments.(TIF)

S8 Fig Overexpression of RAD51 is not toxic to *rad54* mutants. (A). Serial dilution spot assay to determine the effect of GAL-*RAD51* and GAL-*rad51I345T* over-expression on *RAD54, rad54∆, rad54-S816A, S817A,* and *rad54-S816D, S817D* strains.
(TIF)

S9 Fig Rad54 mutant outcomes during diploid allelic recombination. (A). Graph representing the total recombination outcomes for *WT, rad54∆, rad54-S816A, S817A, rad54-S816D, S817D, and rad54-D525S, D527S, S816D, S817D.* The bar represents the mean, and the error bars the standard deviation of at least three independent experiments. (B). Graph representing the total recombination outcomes for *WT, sgs1∆, rad54-S816A, S817A sgs1∆, and rad54-S816D, S817D sgs1∆*. The bar represents the mean, and the error bars the standard deviation of at least three independent experiments.
(TIF)

S10 Fig Purification of Rad54 mutant proteins. (A). Representative Coomassie brilliant blue (R250) stained SDS-PAGE illustrating purified versions of GFP-GST-Rad54, GFP-GST-Rad54 S816A/S817A, GFP-GST-Rad54 S816D/S817D, and GFP-GST-Rad54 D525S/D527S/S816D/S817D.
(TIF)

S11 Fig *rad54-S816D, S817D* overexpression does not fully rescue repair phenotypes. (A). Serial dilution spot assay for *rad54∆* complemented with *pRS415-RAD54, pRS425-RAD54*, empty vector, *pRS415-rad54-S816D, S817D*, and *pRS425-rad54-S816D, S817D*. Strains were tested with no MMS, 0.001%, 0.003%, and 0.01% MMS. (B). Schematic diagram illustrating the system used to test the repair of a double strand break from an ectopic donor. (C). A graph representing the survival percentage for colonies recovering from a double strand break for *pRS415-RAD54, pRS425-RAD54*, empty vector, *pRS415-rad54-S816D, S817D*, and *pRS425-rad54-S816D, S817D.* The bar represents the mean, and the error bars represent the standard deviation of the data.
(TIF)

S12 Fig Rad54 S816D/S817D fails to stabilize D-loops *in vitro.* (A). Graph representing the estimated number of Rad54 and Rad54 S816D/S817D molecules bound to the PSC with (N = 96 and N = 271, respectively) and without (N = 51 and N = 24, respectively) continuous buffer flow. In the buffer flow-plus conditions, the molecules were incubated for 5 minutes in the absence of flow before analysis. The bars represent the mean, and the error bars represent the standard deviation of the data. (B). A graph representing the intensity of Atto647N-90 mer ssDNA for PSCs with Rad54 and Rad54 S816D/S817D with (N = 101, N = 205, respectively) and without (N = 55, N = 24, respectively) continuous buffer flow. The bar represents the mean, and the error bars represent the standard deviation of the data. (C). A representative gel for an *in vitro* D-loop formation experiment for Rad54, Rad54 S816A/S817A, and Rad54 S816D/S817D. The different lanes represent a time course, and the band’s disappearance is consistent with previously described D-loop disassembly.
(TIF)
