# Supplementary material for: Potential Rad54 separation of function mutation highlights unique roles during homologous recombination
Source: PLoS Genet. 2026 Apr 27;22(4):e1012136. doi: 10.1371/journal.pgen.1012136 (PMC13138755; doi:10.1371/journal.pgen.1012136)
Supplement: S1 Table — (PDF) [file pgen.1012136.s001.pdf]

Supporting Table 1
Strains used in the study

Strains	Genotype	Source or reference
BY4741 (WT)		Dharmacon
BY4741-*rad54*Δ	*rad54*Δ	Dharmacon
BY4741	*RAD54::rad54S816A-KANMX*	This study
BY4741	*RAD54::rad54S817A-KANMX*	This study
BY4741	*RAD54::rad54S816A,S817A-KANMX*	This study
BY4741	*RAD54::rad54S816D-KANMX*	This study
BY4741	*RAD54::rad54S817D-KANMX*	This study
BY4741	*RAD54::rad54S816D,S817D-KANMX*	This study
WDHY5511	*ura3::A-HOcs, lys2::A, trp1::GAL-HO-hphMX, his3D200, can1-100, leu2–3, 112, ade2–1, RAD5*	Piazza et al 2020
JBC423 (WDHY5511)	*rad54::KANMX*	This study
JBC281 (WDHY5511)	*RAD54::RAD54-KANMX*	This study
JBC367 (WDHY5511)	*RAD54::rad54S816A,S817A-KANMX*	This study
JBC369 (WDHY5511)	*RAD54::rad54S816D,S817D-KANMX*	This study
JBC380 (WDHY5511)	*sgs1::HISIII*	This study
JBC388 (WDHY5511)	*RAD54::rad54S816A,S817A-KANMX, sgs1::HISIII*	This study
JBC478 (WDHY5511)	*RAD54::rad54S816D,S817D-KANMX, sgs1::HISIII*	This Study
JBC545 (WDHY5511)	*RAD54::rad54D525S, D527S, S816D,S817D-KANMX*	
TGI354	*TGI 354 JKM 146 (arg5,6::MATa::HPH)*	Ira et al 2003
JBC425 (TGI354)	*rad54::KANMX*	This study
JBC357 (TGI354)	*RAD54::RAD54-KANMX*	This study
JBC359 (TGI354)	*RAD54::rad54S816A,S817A-KANMX*	This study
JBC361 (TGI354)	*RAD54::rad54S816D,S817D-KANMX*	This study
Protease deficient yeast strain	*MATα leu2 trp1 ura3–52 prb1–1122 his3::pGAL1–GAL*	Crickard et al 2020
11C (LYS2202)	*MATα ade2-I lys2:GAL-ISCEI his3:HphMX4*	Mazon et al. 2010
15D (LYS2205)	*MATa ade2-n his3:NatMX4 met22:klURA3*	Mazon et al. 2010
JBC106	*11C-RAD54::RAD54-KanMX*	Keymakh et al 2022
JBC105	*15D-RAD54::RAD54-KanMX*	Keymakh et al 2022
JBC413	*11C-RAD54::rad54S816AS817A-KanMX*	This Study

JBC414	*15D-RAD54::rad54S816AS817A-KanMX*	This Study
JBC479	*11C-RAD54::rad54S816DS817D-KanMX*	This Study
JBC417	*15D-RAD54::rad54S816DS817D-KanMX*	This Study
JBC 434	*11C-RAD54::RAD54-KanMX sgs1::HIS3*	This Study
JBC 436	*15D-RAD54::RAD54-KanMX sg1::HIS3*	This Study
JBC 430	*11C-RAD54::rad54S816AS817A-KanMX sgs1::HIS3*	This Study
JBC 432	*15D-RAD54::rad54S816AS817A-KanMX sgs1::HIS3*	This Study
JBC522	*11C-RAD54::rad54S816DS817D-KanMX sgs1::HIS3*	This Study
JBC444	*15D-RAD54::rad54S816DS817D-KanMX sgs1::HIS3*	This Study
JBC554	*11C-RAD54::rad54D525S, D527S, S816D,S817D-KANMX*	This Study
JBC556	*15D-RAD54::rad54D525S, D527S, S816D,S817D-KANMX*	This Study
JBC551	*11C- RAD54::KANMX (rad54Δ)*	This Study
JBC552	*15D- RAD54::KANMX (rad54Δ)*	This Study
JBC514	*W303 MATa rad54::KanMX*	This Study
**Diploids**		
JBC106xJBC105		
JBC413xJBC414		This Study
JBC479xJBC417		This Study
JBC434xJBC436		This Study
JBC430x JBC432		This Study
JBC444xJBC522		This Study
JBC554xJBC556		This Study
JBC551xJBC552		This Study
