# Supplementary material for: Potential Rad54 separation of function mutation highlights unique roles during homologous recombination
Source: PLoS Genet. 2026 Apr 27;22(4):e1012136. doi: 10.1371/journal.pgen.1012136 (PMC13138755; doi:10.1371/journal.pgen.1012136)
Supplement: S2 Table — (PDF) [file pgen.1012136.s002.pdf]

Supporting Table 2
Plasmid in the study

Backbone	Construction	Source
pRS305	*RAD54-KANMX*	This study
pRS305	*rad54S816A-KANMX*	This study
pRS305	*rad54S816D-KANMX*	This study
pRS305	*rad54-S816AS817A-KANMX*	This study
pRS305	*rad54-S816DS817D-KANMX*	This study
pRS305	*rad54-D525S,D527S,S816DS817D-KANMX*	This study
pRS415	*RAD54*	Crickard et al. 2020
pRS415	*rad54-S816A*	This study
pRS415	*rad54-S816D*	This study
pRS415	*rad54-S816AS817A*	This study
pRS415	*rad54-S816DS817D*	This study
pRS415	*rad54-T85A*	This study
pRS415	*rad54-T85E*	This study
pRS415	*rad54-T132A*	This study
pRS415	*rad54-T132E*	This study
pRS415	*rad54-T155A*	This study
pRS415	*rad54-T155E*	This study
pRS415	*rad54-S214A*	This study
pRS415	*rad54-S214D*	This study
pRS415	*rad54-T231A*	This study
pRS415	*rad54-T231E*	This study
pRS415	*rad54-S303A*	This study
pRS415	*rad54-S303D*	This study
pRS415	*rad54-S318A*	This study
pRS415	*rad54-S318D*	This study
pRS415	*rad54-D525A*	This study
pRS415	*rad54-D525K*	This study
pRS415	*rad54-D525S*	This study
pRS415	*rad54-D525N*	This study
pRS415	*rad54-D525A, D527A*	This study
pRS415	*rad54-D525K, D527K*	This study
pRS415	*rad54-D525S, D527S*	This study
pRS415	*rad54-D525N, D527N*	This study
pRS415	*rad54-D525A, D527A, S816A, S817A*	This study
pRS415	*rad54-D525K, D527K, S816A, S817A*	This study
pRS415	*rad54-D525S, D527S, S816A, S817A*	This study
pRS415	*rad54-D525N, D527N, S816A, S817A*	This study

pRS415	*rad54-D525A, D527A, S816D, S817D*	This study
pRS415	*rad54-D525K, D527K, S816D, S817D*	This study
pRS415	*rad54-D525S, D527S, S816D, S817D*	This study
pRS415	*rad54-D525N, D527N, S816D, S817D*	This study
