# Supplementary material for: Potential Rad54 separation of function mutation highlights unique roles during homologous recombination
Source: PLoS Genet. 2026 Apr 27;22(4):e1012136. doi: 10.1371/journal.pgen.1012136 (PMC13138755; doi:10.1371/journal.pgen.1012136)
Supplement: S3 Table — (PDF) [file pgen.1012136.s003.pdf]

Supporting Table 3

Oligos used in the study

Name	Sequence	Purpose
90-mer DNA	Atto647N-GATGTTCTGCTGGATATGCACTTTTCCGGGCTGACGTACACCGTGCTCAGCCTGTTTTTCAGCGATCCGGATATGCATCCGCTGGATTTC	Single Molecule imaging
olWDH1760	CAGCGGGCTTGCAGAAGTTG	To amplify genomic DNA at *ARG4*
olWDH1761	GGCCAATTAGTTCACCAAGACG	To amplify genomic DNA at *ARG4*
olWDH1766	GTTTCAGCTTTCCGCAACAG	To quantify DSB induction
olWDH1767	GGCGAGGTATTGGATAGTTCC	To quantify DSB induction
olWDH2009	CACCACTTTGCCATTCAACAC	To amplify at the downstream site of elongation
olWDH2010	TGCTCGGAGATTACCGAATC	To amplify at upstream site of the DSB, used with olWDH2009 to quantify DLE signal
olWDH2011	TGCGAGGTTTTCTTGGTCAG	Used with olWDH2009 to quantify *Hin*dIII recognition site restoration
olWDH2012	CGAGGCATATTTATGGTGAAGG	Used with olWDH2010 to measures *Hin*dIII recognition site restoration
olWDH2052	ATGTGCCTTCCTACCGCTC	To quantify intramolecular ligation efficiency of *Hin*dIII-derived fragments
olWDH2053	TCAAGCGTGGTTACATTCCTTAC	To quantify intramolecular ligation efficiency of *Hin*dIII-derived fragments
olWDH2007	TCTGCTCGGAGATTACCGAATCAAAAAAATTTCAAAGAAACCGGAATCAAAAAAAAGAACAAAAAAAAAAAAGATGAATTGAAAAGCTTTATGGACCGAC	To restore *Hin*dIII site

olWDH2046	AATCTTTGTGAAGCTTCGCAAGTATTCATTTTAGACCCATGGTGGAACCCTAGTGTTGAATGGCAAAGTGGTGATAGAGTTCATAGAATTGGTCAGTAT	To restore *Hin*dIII site
olWDH1762	ACTTCGAATTTCGGCACTTC	To quantify intramolecular ligation efficiency of *Eco*RI-derived fragments
olWDH1763	CGATGAAACGTTAAGTGACCAC	To quantify intramolecular ligation efficiency of *Eco*RI-derived fragments
olWDH1764	AGAGCGGTCAGTAGCAATCC	To amplify at the upstream of DSB
olWDH1765	CACACGCGAAAAACCGCC	To amplify at the upstream of the donor DNA, used with olWDH1764 to quantify DLC signal
olWDH2019	CTTTAACCGGACGCTCGA	To quantify psoralen crosslinking efficiency
olWDH2020	TTGAGTTTATTGCTGCCGTC	To quantify psoralen crosslinking efficiency
olWDH1768	AGGAGCACAGACTTAGATTGG	Used with olWDH1764 to measure *Eco*RI recognition site restoration
olWDH1770	CGAAATCATCTTCGGTTAAATCCAAAACGGCAGAAGCCTGAATGAAACATATGAACCAATTGGAGGACGTCAATGAATTCTGGGGATCCATTGCATTTTT	To restore *Eco*RI site
