# Supplementary figures and images for: Potential Rad54 separation of function mutation highlights unique roles during homologous recombination
Source: PLoS Genet. 2026 Apr 27;22(4):e1012136. doi: 10.1371/journal.pgen.1012136 (PMC13138755; doi:10.1371/journal.pgen.1012136)

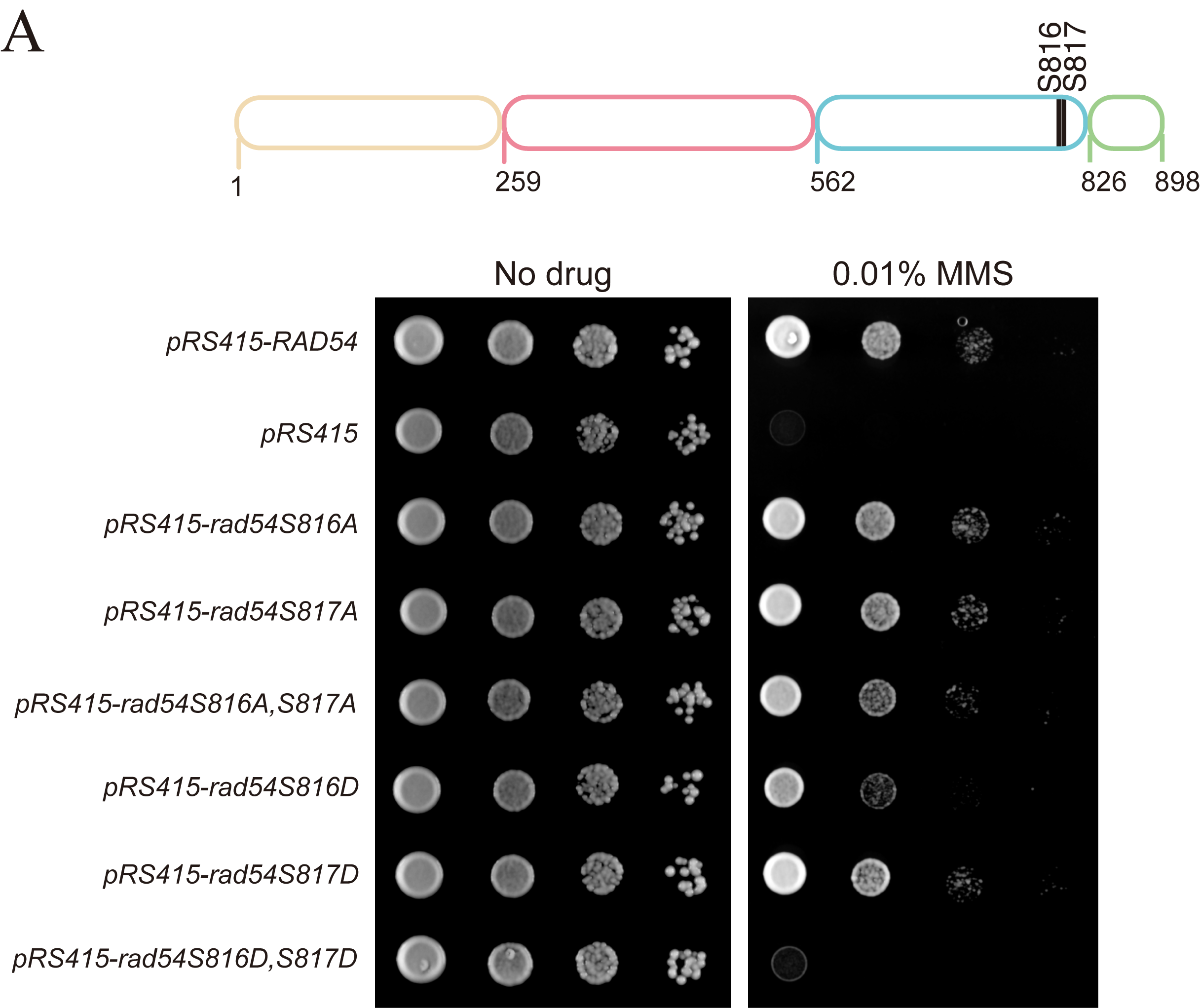

Supplement: S1 Fig — (A). Serial dilution spot assay to determine the sensitivity of yeast strains with single amino acid substitutions at Rad54 S816 and S817 or double mutations at the same positions. Serine was mutated to either Alanine or Aspartic acid. (TIF) [file pgen.1012136.s004.tif]

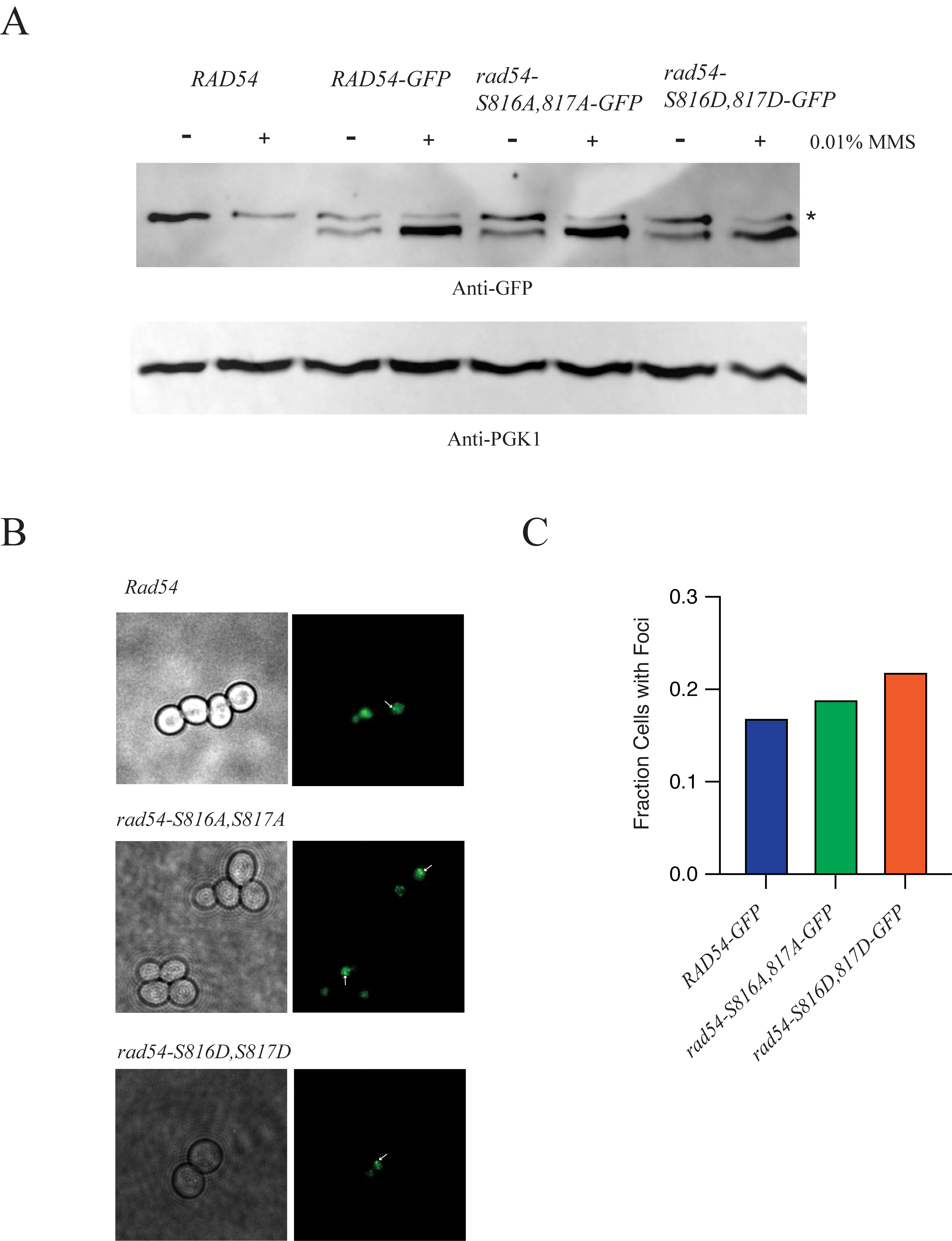

Supplement: S2 Fig — (A). Representative western blot illustrating the expression of RAD54-GFP, rad54-S816A, S817A-GFP, and rad54-S816D, S817D-GFP with and without 0.01% MMS. PGK1 is used as a loading control. The Asterix represents a non-specific band. (B). Fluorescent microscope images illustrating that RAD54-GFP, rad54-S816A, S817A-GFP, and rad54-S816D, S817D-GFP form foci in response to MMS treatment. (C). Bar graph quantifying the percentage of cells that form foci in response to MMS treatment. (TIF) [file pgen.1012136.s005.tif]

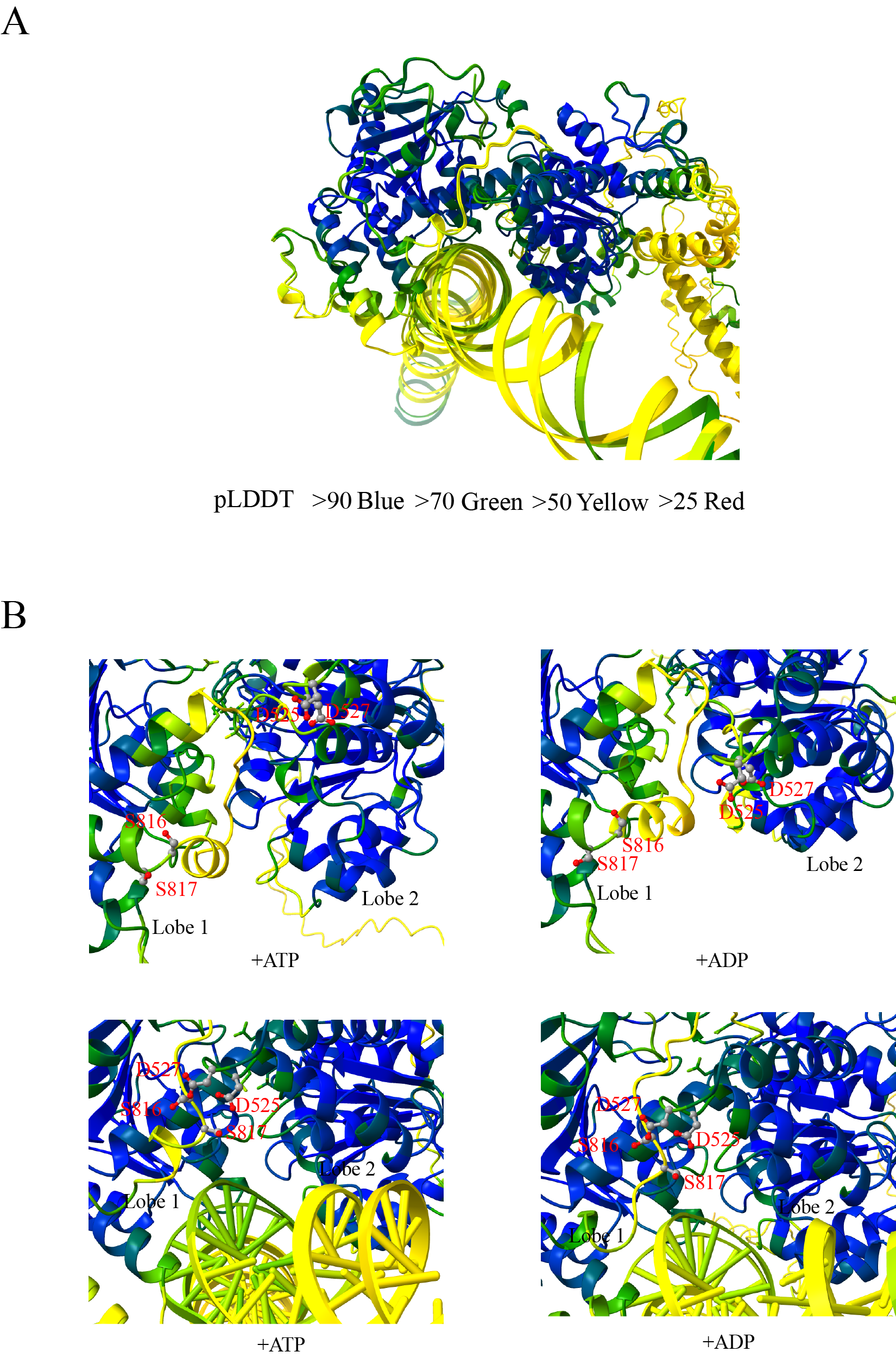

Supplement: S3 Fig — (A). AlphaFold3 model for S. cerevisiae Rad54 bound to dsDNA, colored for confidence with the pLDDT formation. Structures predicted with ATP and ADP are overlayed. (B). AlphaFold predictions for Rad54 without DNA and with ATP (Top, Left), Rad54 without DNA with ADP (Top, Right), AlphaFold predictions for Rad54 with DNA and with ATP (Bottom, Left), Rad54 with DNA with ADP (Bottom, Right). Residues D525, D527, S816, and S817 are highlighted. All structures are color coded with pLDDT colors as listed in the figure legend in A. (TIF) [file pgen.1012136.s006.tif]

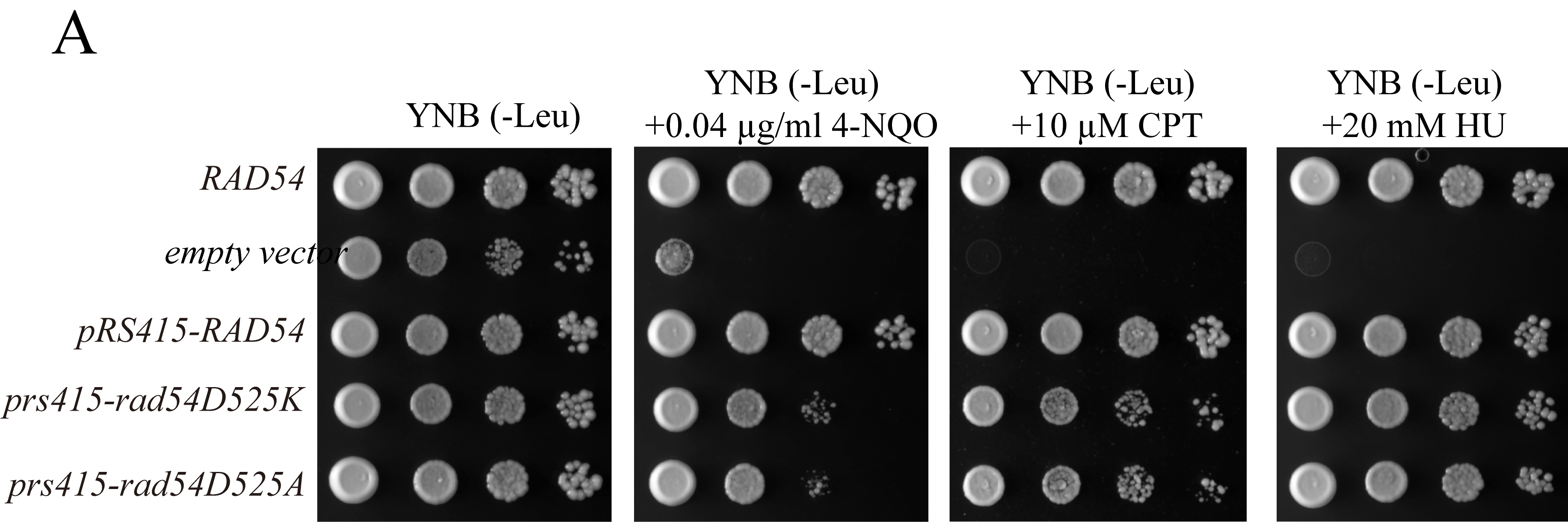

Supplement: S4 Fig — (A). Yeast spot assays testing the ability of rad54-D525A and rad54-D525K to complement 4-NQO, CPT, and HU sensitivity. (TIF) [file pgen.1012136.s007.tif]

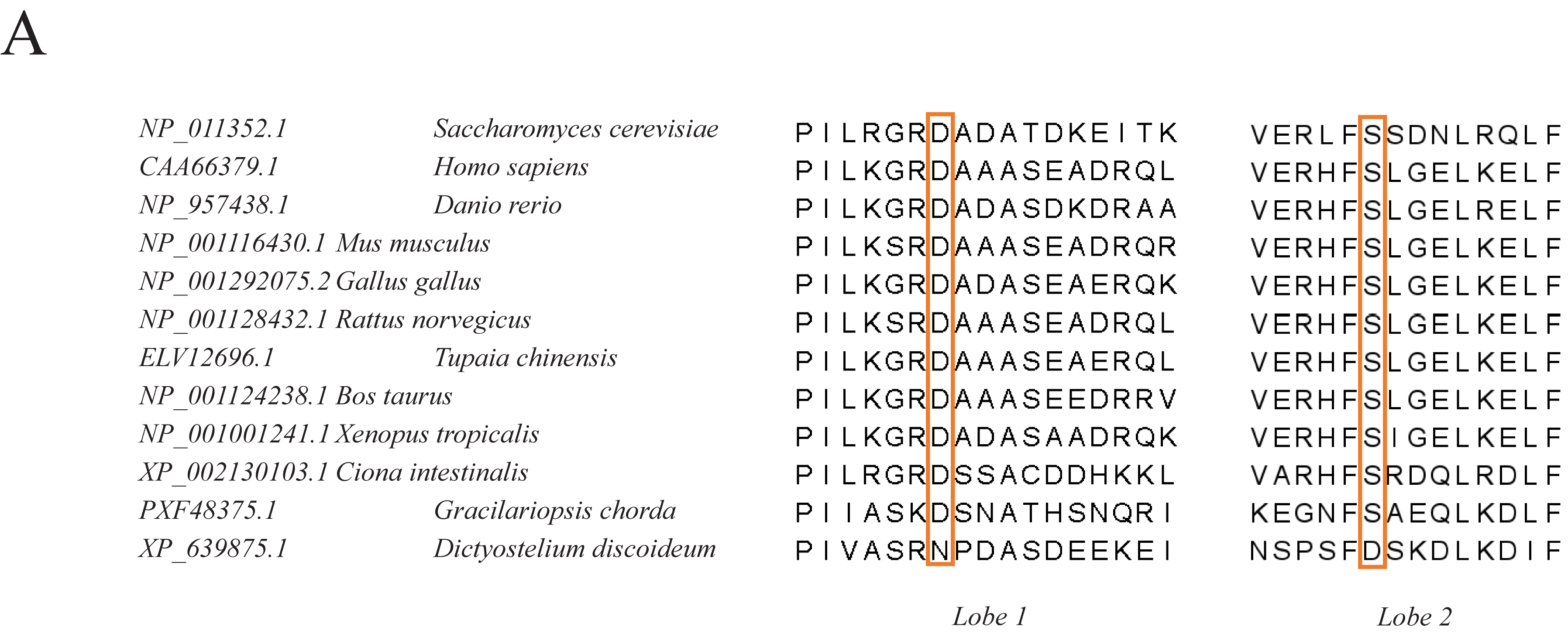

Supplement: S5 Fig — (A). An excerpt from the Rad54 multiple sequence alignment. Shown are the two interacting regions on lobes 1 and 2, respectively. Included in this alignment in Dictyostelium discoideum, which has a naturally occurring Aspartic acid in place of Serine on lobe 2. (TIF) [file pgen.1012136.s008.tif]

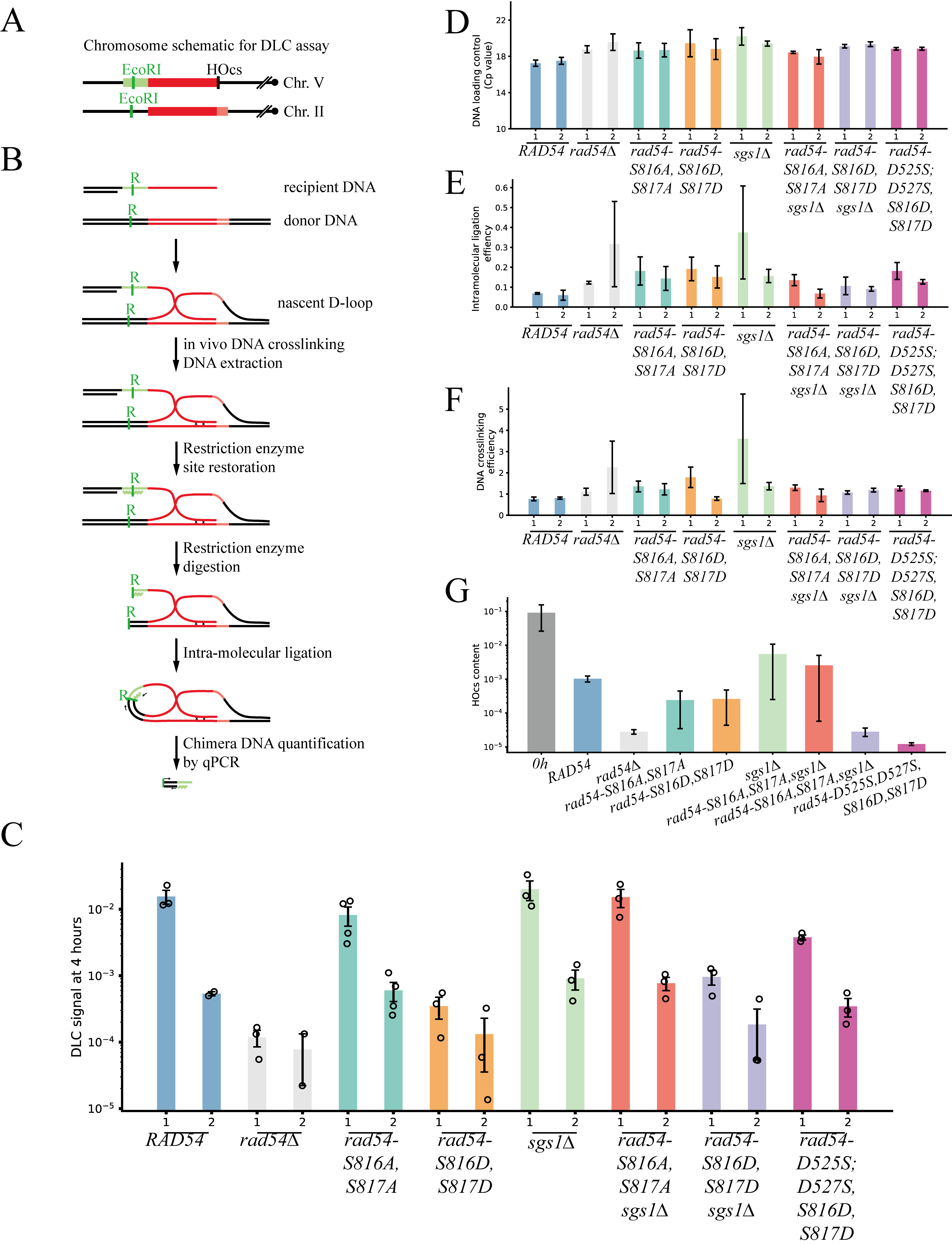

Supplement: S6 Fig — (A). Chromosome schematic for the DLC assay. (B). Workflow for the DLC assay. (C). DLC signal at 4 hours. Column set 1 shows results with a hybrid oligo added; column set 2 shows results without adding a hybrid oligo. (D). DNA loading control (Cp values for amplicons at ARG4) for RAD54; rad54∆; rad54-S816A, S817A; rad54-S816D, S817D sgs1∆; rad54-S816A, S817A sgs1∆; rad54-S816D, S817D sgs1∆; and rad54-D525S, D527S, S816D, S817D. Columns: (1) 4 hours with hybrid oligo; (2) 4 hours without hybrid oligo. (E). Intramolecular ligation efficiency calculated as Intramolecularligationamplificationefficiency−Cp(ligation)ARG4amplificationefficiency−Cp(ARG4). Columns as in (D). (F). Crosslinking efficiency calculated as ssDNAamplificationefficiency−Cp(ssDNA)ARG4amplificationefficiency−Cp(ARG4). Columns as in (D). (G). DNA content at HO cut sites,calculated as HOcutsiteamplificationeffieciency−Cp(HOcs)ARG4amplificationefficiency−Cp(ARG4). The “0h” value represents the mean across all available 0-hour time point groups from all genotypes with a hybrid oligo added. Other columns represent DNA content at HO cut sites at 4-hour timepoint. (TIF) [file pgen.1012136.s009.tif]

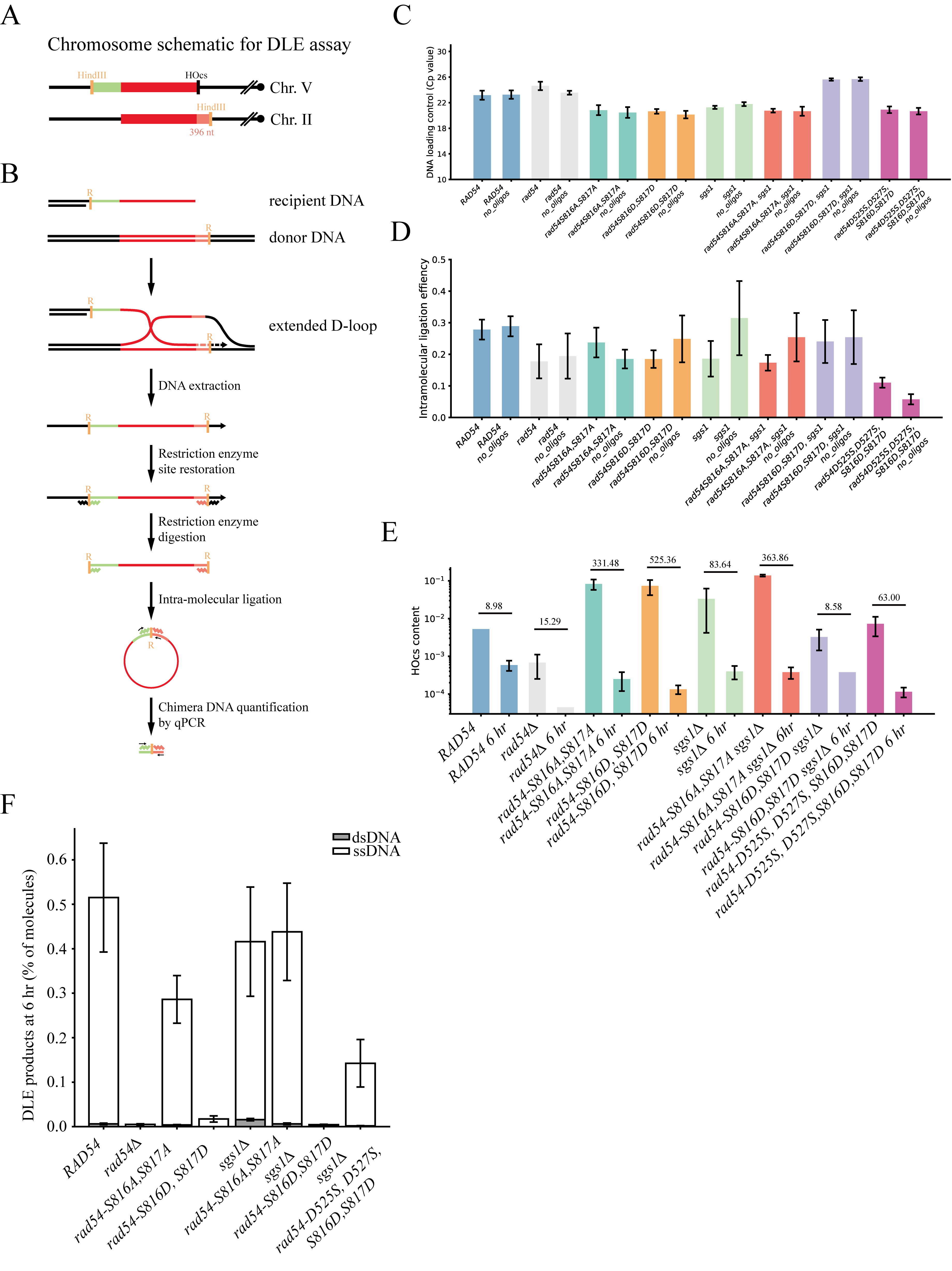

Supplement: S7 Fig — (A). Chromosome schematic for the DLE assay. (B). Workflow for the DLE assay. (C). DNA loading control (Cp values for amplicons at ARG4). For RAD54; rad54∆; rad54-S816A, S817A; rad54-S816D, S817D; sgs1∆; rad54-S816A, S817A sgs1∆; rad54-S816D, S817D sgs1∆; and rad54-D525S, D527S, S816D, S817D. (D). Intramolecular ligation efficiency calculated as Intramolecularligationamplificationefficiency−Cp(ligation)ARG4amplificationefficiency−Cp(ARG4) for allgenotypes. (E). DNA content at HO cut sites HOcutsiteamplificationeffieciency−Cp(HOcs)ARG4amplificationefficiency−Cp(ARG4) for all genotypes at 0-hour and 6-hour time points with hybrid oligos added. Numbers above the bars indicate the reduction in DNA content at the HO cut site after 6 hours. (F). Extension products at 6 hours: single-stranded (ssDNA; DLE signal when both hybrid oligos were added) and double-stranded (dsDNA; DLE signal when no hybrid oligos were added) for all genotypes. The error bars represent the standard error measurement of at least three independent experiments. (TIF) [file pgen.1012136.s010.tif]

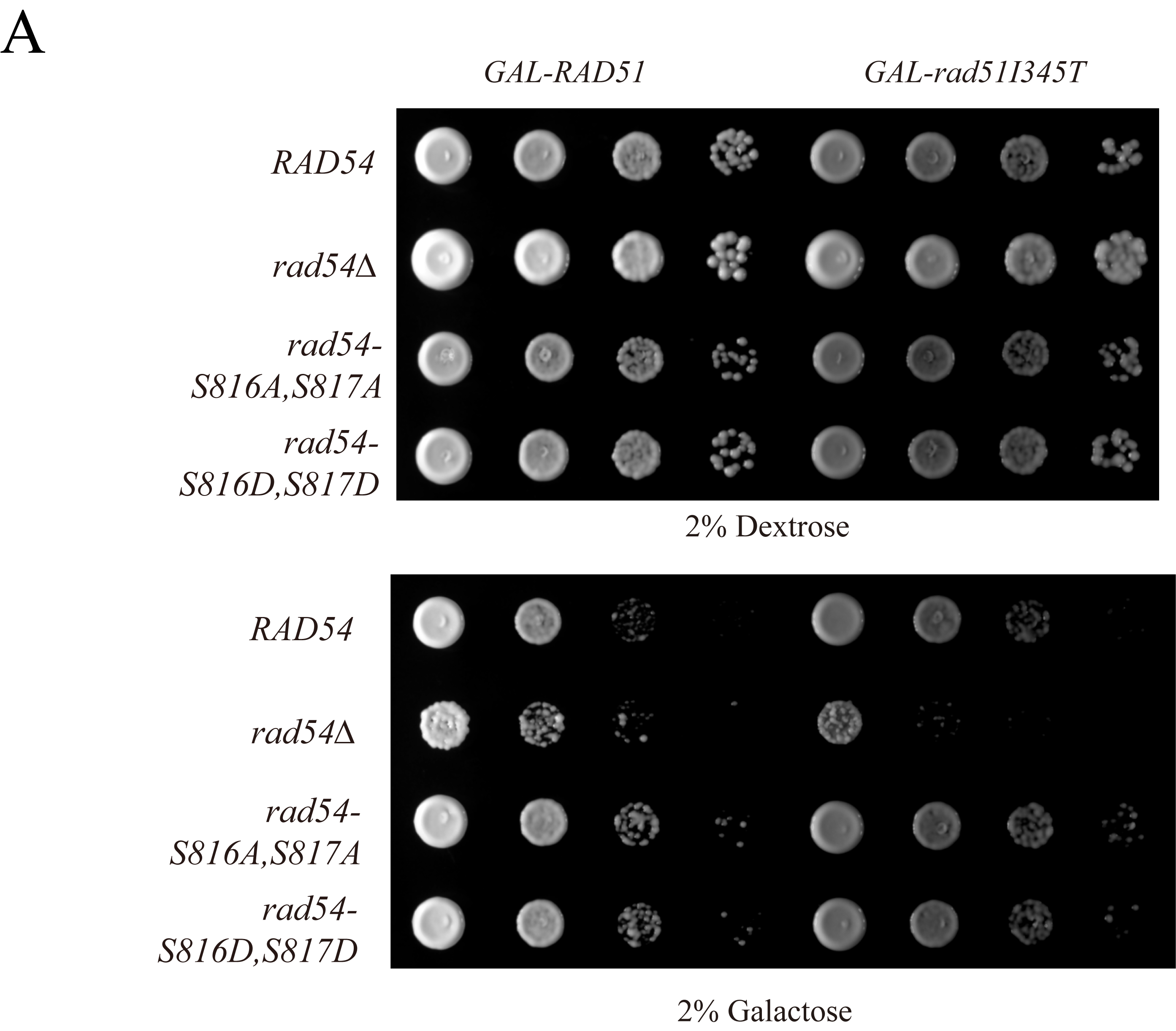

Supplement: S8 Fig — (A). Serial dilution spot assay to determine the effect of GAL-RAD51 and GAL-rad51I345T over-expression on RAD54, rad54∆, rad54-S816A, S817A, and rad54-S816D, S817D strains. (TIF) [file pgen.1012136.s011.tif]

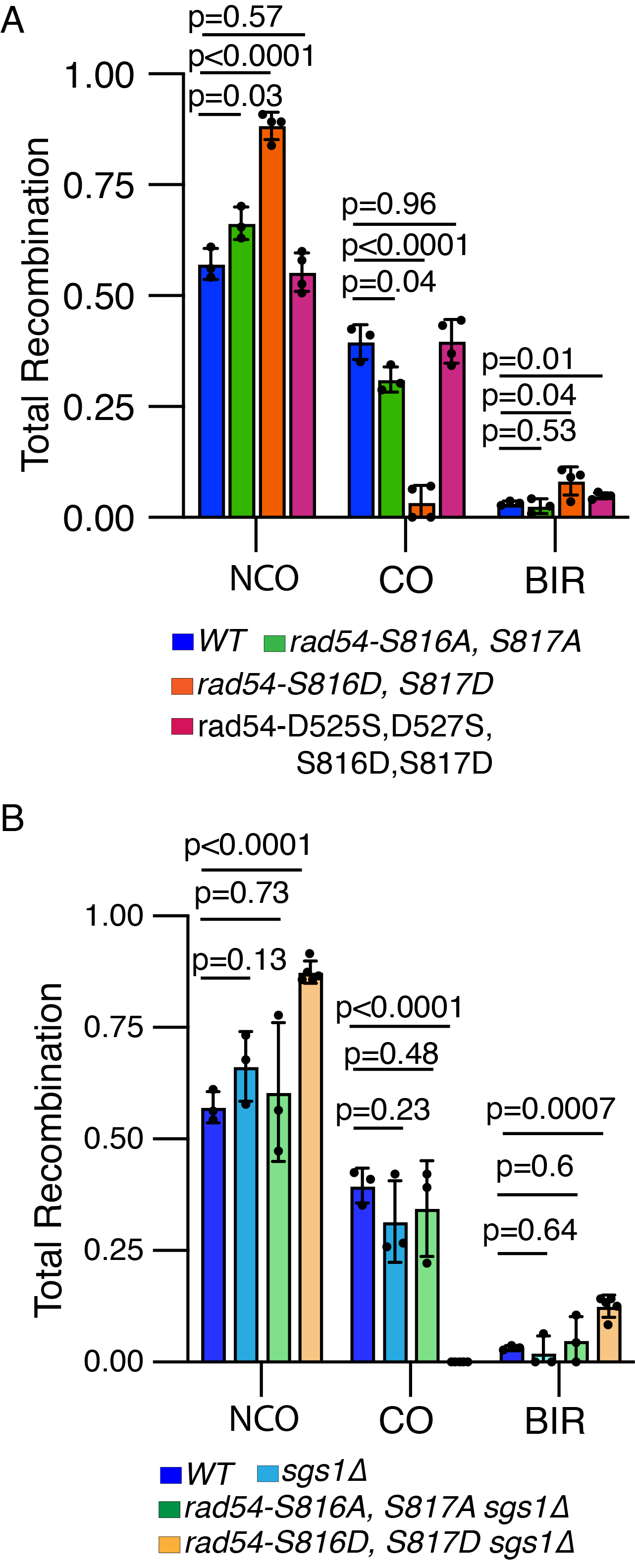

Supplement: S9 Fig — (A). Graph representing the total recombination outcomes for WT, rad54∆, rad54-S816A, S817A, rad54-S816D, S817D, and rad54-D525S, D527S, S816D, S817D. The bar represents the mean, and the error bars the standard deviation of at least three independent experiments. (B). Graph representing the total recombination outcomes for WT, sgs1∆, rad54-S816A, S817A sgs1∆, and rad54-S816D, S817D sgs1∆. The bar represents the mean, and the error bars the standard deviation of at least three independent experiments. (TIF) [file pgen.1012136.s012.tif]

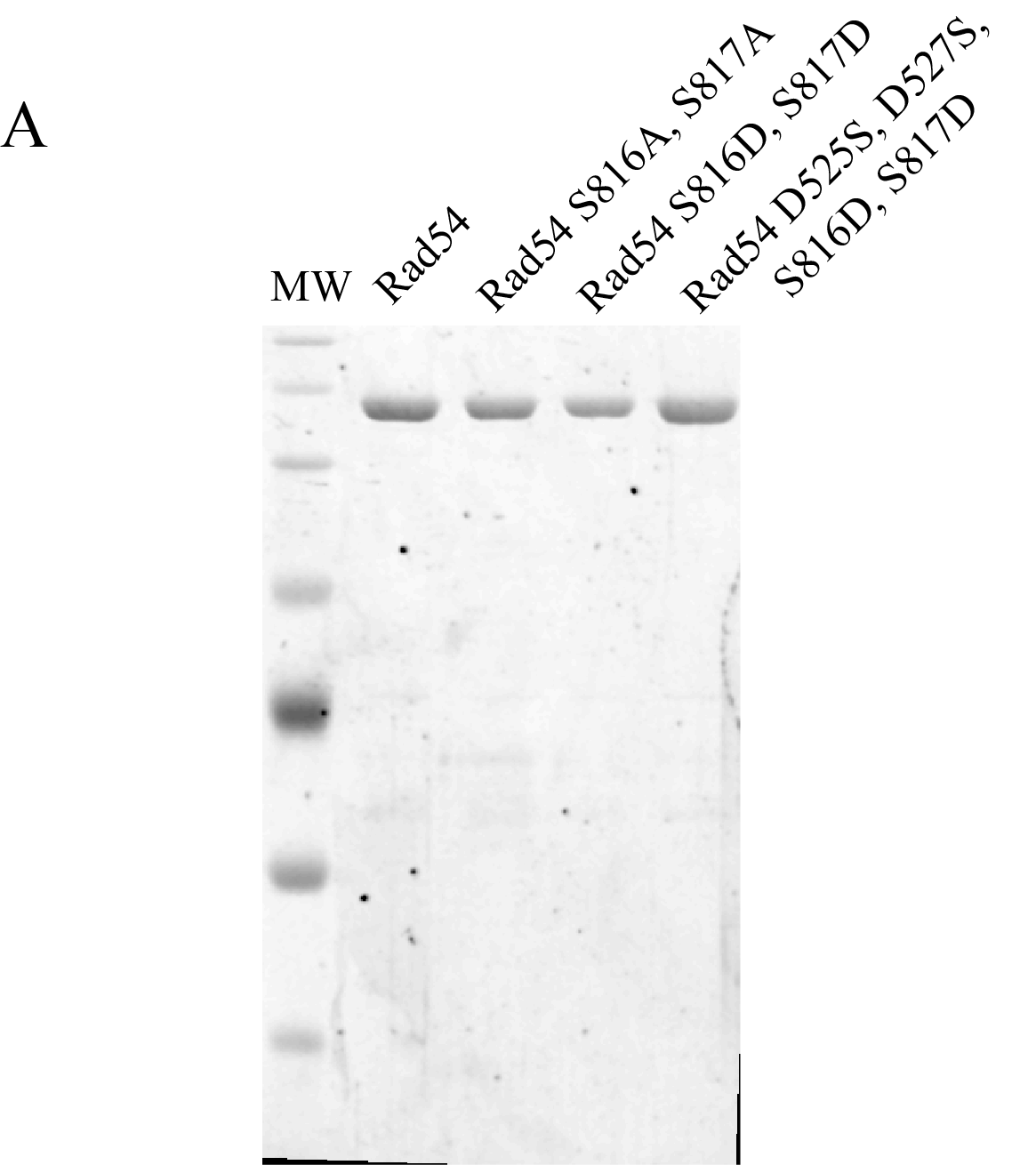

Supplement: S10 Fig — (A). Representative Coomassie brilliant blue (R250) stained SDS-PAGE illustrating purified versions of GFP-GST-Rad54, GFP-GST-Rad54 S816A/S817A, GFP-GST-Rad54 S816D/S817D, and GFP-GST-Rad54 D525S/D527S/S816D/S817D. (TIF) [file pgen.1012136.s013.tif]

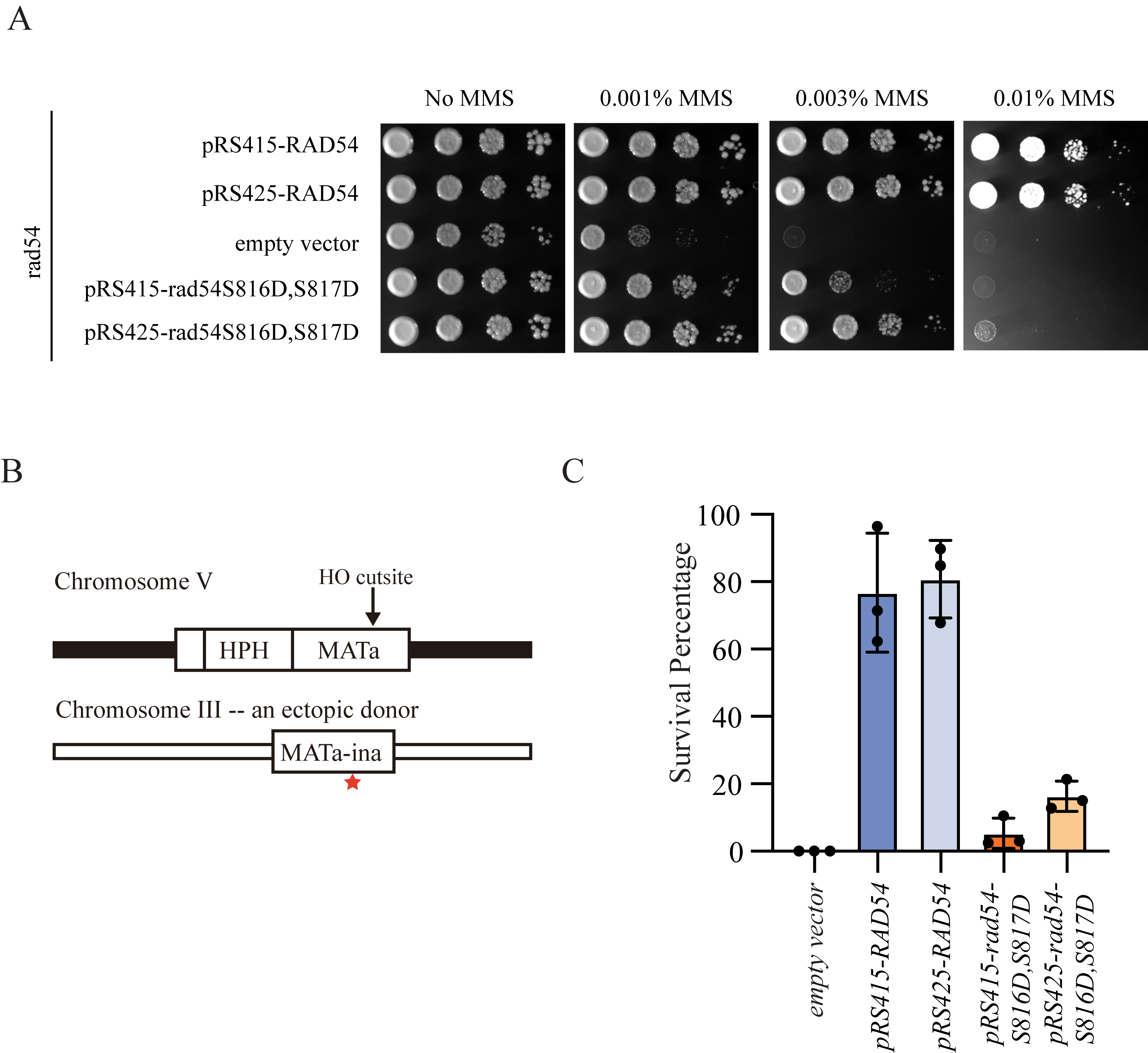

Supplement: S11 Fig — (A). Serial dilution spot assay for rad54∆ complemented with pRS415-RAD54, pRS425-RAD54, empty vector, pRS415-rad54-S816D, S817D, and pRS425-rad54-S816D, S817D. Strains were tested with no MMS, 0.001%, 0.003%, and 0.01% MMS. (B). Schematic diagram illustrating the system used to test the repair of a double strand break from an ectopic donor. (C). A graph representing the survival percentage for colonies recovering from a double strand break for pRS415-RAD54, pRS425-RAD54, empty vector, pRS415-rad54-S816D, S817D, and pRS425-rad54-S816D, S817D. The bar represents the mean, and the error bars represent the standard deviation of the data. (TIF) [file pgen.1012136.s014.tif]

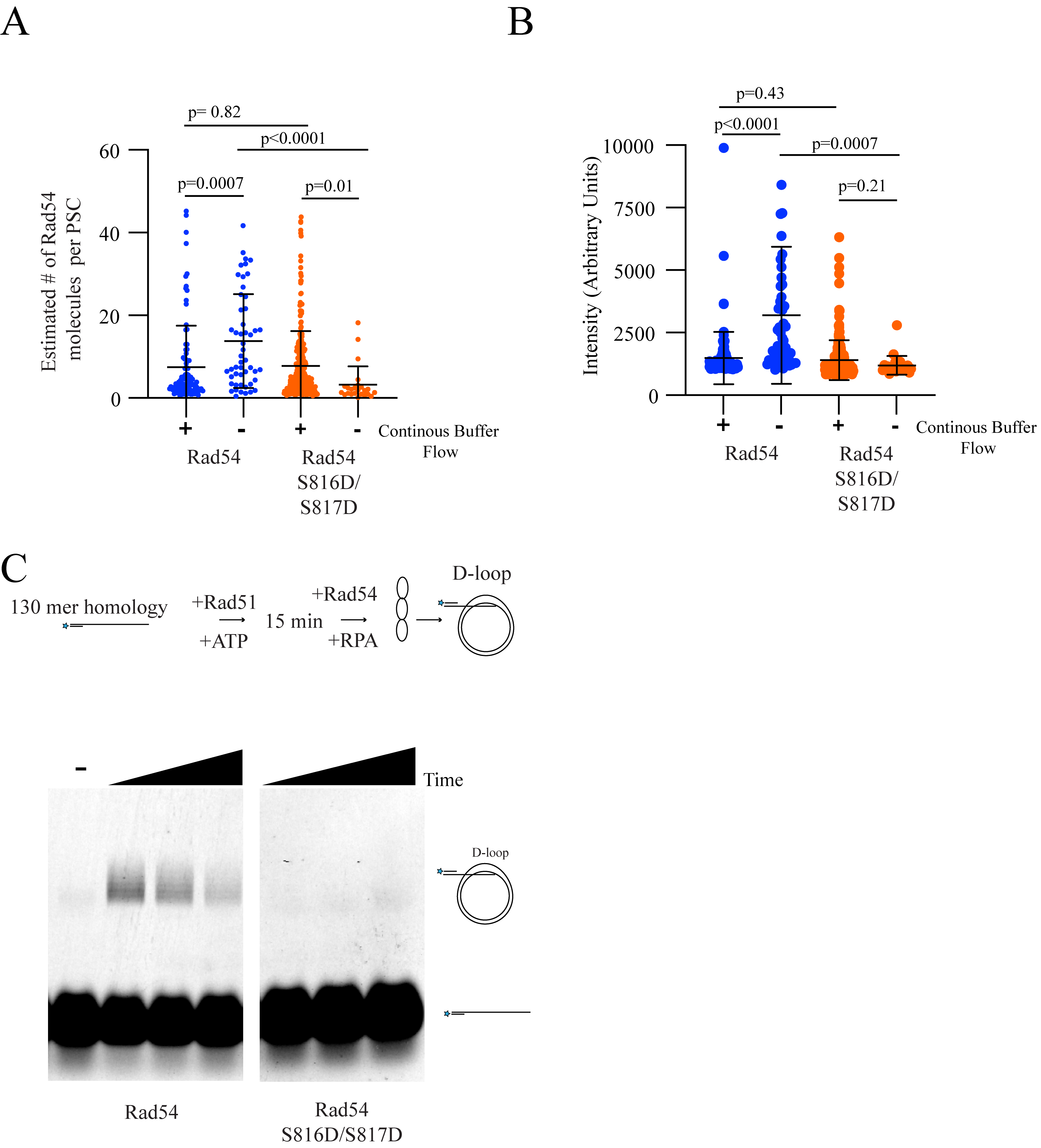

Supplement: S12 Fig — (A). Graph representing the estimated number of Rad54 and Rad54 S816D/S817D molecules bound to the PSC with (N = 96 and N = 271, respectively) and without (N = 51 and N = 24, respectively) continuous buffer flow. In the buffer flow-plus conditions, the molecules were incubated for 5 minutes in the absence of flow before analysis. The bars represent the mean, and the error bars represent the standard deviation of the data. (B). A graph representing the intensity of Atto647N-90 mer ssDNA for PSCs with Rad54 and Rad54 S816D/S817D with (N = 101, N = 205, respectively) and without (N = 55, N = 24, respectively) continuous buffer flow. The bar represents the mean, and the error bars represent the standard deviation of the data. (C). A representative gel for an in vitro D-loop formation experiment for Rad54, Rad54 S816A/S817A, and Rad54 S816D/S817D. The different lanes represent a time course, and the band’s disappearance is consistent with previously described D-loop disassembly. (TIF) [file pgen.1012136.s015.tif]
